# Spot on: A Laser Micromachining-Based Approach to Improve Dried Matrix Spot Preparation with Proof-of-Principle Analytical Demonstrations Using Ambient Ionization Mass Spectrometry

**DOI:** 10.3390/mi17050559

**Published:** 2026-04-30

**Authors:** Daniel O. Reddy, Malek Hassan, Jonathan O. Graham, Jared Viggers, Katherine E. Williams, Randy E. Ellis, Thomas R. Covey, Jacob T. Shelley, Richard D. Oleschuk

**Affiliations:** 1 Department of Chemistry, Queen’s University, Kingston, ON K7L 3N6, Canada; daniel.reddy@queensu.ca (D.O.R.); malek.hassan@queensu.ca (M.H.); 2 Department of Chemistry and Chemical Biology, Rensselaer Polytechnic Institute, Troy, NY 12180, USA; grahaj8@rpi.edu (J.O.G.); viggej@rpi.edu (J.V.); shellj@rpi.edu (J.T.S.); 3 School of Computing, Queen’s University, Kingston, ON K7L 2N8, Canada; 17kew2@queensu.ca (K.E.W.); ellis@queensu.ca (R.E.E.); 4 SCIEX, Concord, ON L4K 4V8, Canada; tom.covey@sciex.com

**Keywords:** dried matrix spots, dried blood spots, dried saliva spots, dried urine spots, laser micromachining, micropatterning, paper microfluidics, ambient ionization mass spectrometry

## Abstract

The use of dried matrix spots (DMSs) has recently re-emerged as a useful sample storage technique and analytical platform along with the increased adoption of and general preference for ambient ionization mass-spectrometric methods. However, challenges associated with precise liquid confinement and sample targeting persist. In this paper, we present a laser micromachining-based approach to prepare DMSs on hydrophobic paper substrates that include visual recognition elements, or reticles, around surface energy traps (SETs). This targeted DMS substrate is combined with direct mass spectrometric analyses, namely liquid microjunction–surface sampling probe–mass spectrometry (LMJ-SSP-MS) and flowing atmospheric-pressure afterglow–mass spectrometry (FAPA-MS). With the laser-based micromachining approach, DMSs flanked by crosshairs for enhanced visualization are prepared on SETs as small as 0.55 mm in diameter, which offers an approximately 12-fold reduction in size compared to traditional DMS preparations. The DMSs prepared on these targeting SETs are demonstrated with the detection of caffeine in model aqueous and artificial urine solutions using LMJ-SSP-MS and FAPA-MS, respectively. With further refinement, this approach could be automated using computer vision and robotics to broaden the scope of DMSs and improve the analytical workflow.

## 1. Introduction

Dried matrix spot(s) [DMS(s)] [1,2,3,4], also referred to as dried (sample) spots [D(S)Ss] [5,6,7], are gaining attention and traction as a clinically [8,9] useful sampling tool and ultimately as a sample preparation/storage technique across various fields with applications ranging from environmental science [10,11,12] to toxicology [13,14,15]. While the DMS technique does not necessarily require that a liquid sample be biological in origin, the most common implementations of DMSs include biological liquids, or biofluids [16], like blood [dried blood spots (DBSs)] [17,18,19], saliva [dried saliva spots (DSSs), sometimes abbreviated as DSaSs [20] to avoid confusion with dried sample spots (DSSs)] [21,22,23], and urine [dried urine spots (DUSs)] [24,25,26]. At its core, the DMS technique is simply the application [27] of a liquid sample onto a storage substrate, which is often filter paper [28,29,30]. Once a volume of liquid sample has been applied to the storage substrate and dried after some time depending on the ambient conditions and type/volume of liquid, a DMS is considered to have been successfully prepared; this spotting step is generally performed manually by pipetting [31,32,33] or by directly touching the storage substrate to the source liquid that is to be sampled [34,35]. As a note, while there are detailed reports of fully automated DMS methods [24,31,32,36,37], such automation is in regard to the workflow post-DMS preparation (i.e., the DMS processing and analysis steps) not the DMS preparation step itself where the liquid is spotted onto the substrate [though this distinction is understandable given considerations like small volumes and viscosity associated with (automated) liquid handling [38,39], especially for “challenging” [40,41,42] liquids like blood].

While there have been great strides related to DMSs, especially with respect to scope and analyte quantitation [6,43,44], further work is required to promote the more widespread adoption of DMSs as a versatile approach for sample preparation and storage. Regarding the analysis of DMSs, there are many clinically validated methods across a range of biofluid and analyte combinations (e.g., artificial cerebrospinal fluid containing N[(3R-1-Azabicyclo[2.2.2]oct-3-yl]furo[2,3]pyridine-5-carboxamide [7], saliva with carbamazepine [45], and serum that includes trace elements [46]). Relatedly, these methods often rely on liquid chromatography–mass spectrometry/(mass spectrometry) [LC-MS/(MS] analysis [47], which is viewed as the gold standard [48,49] for quantitative DMS analysis, though other analytical techniques have been also used to both qualitatively [14,49] and (semi-)quantitatively [50,51] analyze DMS.

Taken together, these aforementioned analytical developments have been crucial to DMS advancements and ultimately clinical acceptance. However, an aspect of DMS that is occasionally overlooked is one of the two core components comprising a DMS besides the liquid sample—that is, the paper substrate [20]. Nonetheless, there has indeed been impressive work to bolster and understand the substrate as the workhorse that enables the preparation, processing, and analysis of DMSs, such as silica-coated or otherwise functionalized paper [52,53,54,55], comparisons of commercially available products [56,57], and fundamental analysis of liquid flow [58,59,60,61,62]. Still, while paper is an ideal and renowned substrate for DMSs, especially owing to considerations like lower cost and sustainability [63,64,65,66], paper as a DMS substrate can present certain other challenges due to its inherent properties, namely lateral and transverse diffusion of the liquid sample [3,67,68]. Sample diffusion and its related considerations are well-documented aspects of DMSs [57,69,70,71]. That said, at times, sample diffusion is not necessarily problematic and/or there are ways to mitigate the problems like sample (in)homogeneity and analyte losses posed by such diffusion. For example, in a typical DMS workflow, the liquid sample is spotted onto the substrate and allowed to diffuse and wick via capillary action; the DMS can then be stored for later processing, at which point a hole (or holes) is/(are) punched and removed from the DMS. The resultant disc(s) from the original DMS sample are then digested and extracted, and the solution is analyzed. In such a workflow, while spot inhomogeneity and punch-location irreproducibility are not ideal, the near-completeness of the digestion step mostly compensates for the downsides. Additionally, there have been several reports that seek to better confine the liquid sample during dry-down. For example, Baillargeon et al. (2022) propose a patterned DBS card with four uniform spots, where each spot (~6 mm diameter) functions as a sample well to contain ~10 μL of blood [72]. Relatedly, Kim et al. (2013) use a commercially available plasma extraction card (NoviPlex) to prepare a dried plasma spot for mass-spectrometric analysis [73]; the card serves to spread, separate, and collect approximately 2.5 μL of plasma from a ca. 25 μL of whole blood. Ultimately, the plasma concentrates into a 6.35 mm disc that is extracted and analyzed.

Overall, DMSs are important components of the sample preparation and analysis toolkit. Thereby, in this paper, we seek to address the visual targeting and sample diffusion problems faced by traditional DMS substrates by proposing a laser-micromachined, dual-wetting paper substrate with microfluidic features as fine as 0.55 mm—namely, surface energy trap(s) [SET(s)]. Briefly, SETs are precisely defined regions where the surface energy within the region is higher than the surrounding surface energy [74,75,76]. Importantly, this paper provides an adaptable laser micromachining-based approach to prepare visualization elements around the SET for DMS preparation. Especially with colorless samples on a paper substrate, SETs can be difficult, if not impossible, to visualize and thereby deposit the sample. These SETs, flanked by reticle elements that aid in visualization, trap and confine a droplet superficially on the substate (i.e., resisting both lateral and transverse diffusion to strictly retain the liquid). Combined with material characterizations, we highlight the effectiveness of the hydrophobization and laser-micromachining treatments by demonstrating the effectiveness of the substrate through the use of two ambient ionization mass spectrometry techniques: (1) liquid microjunction–surface sampling probe mass spectrometry (LMJ-SSP-MS) and (2) flowing atmospheric pressure afterglow–mass spectrometry (FAPA-MS). It is important, however, to note that this paper focuses on improvements to DMS substrate design and function, along with analytical compatibility as a proof-of-principle, rather than a fully quantitative (bio)analytical validation.

## 2. Materials and Methods

Fisher Scientific filter paper (150 mm diameter, Cytiva Whatman^®^ Qualitative Grade Plain Circles–Grades 1 and 3) was used (Ottawa, ON, Canada). Aculon^®^ A and AL-A were purchased from Aculon, Inc. (San Diego, CA, USA), and blue, green, red, and yellow food dye solutions (Club House brand) were purchased from a local grocery store. Deionized water (DI H_2_O) (>18.2 MΩ cm) was generated with a Sartorius Stedim Biotech Arium^®^ pro DI water system (Gottingen, Germany), and Fisher Chemical methanol (MeOH) [HPLC grade, (Ottawa, ON, Canada)] was used. Merck KGaA formic acid (FA) (LiChropure^®^, 98–100% for HPLC) was used (Darmstadt, Germany). ACROS Organics rhodamine 6G (R6G) (99%, pure) was used, and Sigma-Aldrich^®^ Allura Red AC (Dye Content 80%) and caffeine (powder, ReagentPlus) were used (St. Louis, MO, USA). Aldrich Indigo (Suspension, dil.) (St. Louis, MO, USA) and Sigma Nile Blue (C.I. 51180; Basic Blue 12) Chloride (Dye Content approx. 90%) (St. Louis, MO, USA) were used. Pickering Laboratories artificial urine (Stabilized) (Mountain View, CA, USA) and Ward’s Science artificial urine (Lab grade) (St. Catharines, ON, Canada) were used. A 1.75 mm diameter PETG 3D printer filament in various colors was purchased from various commercial sources, and Prusa Research 3D printers (Original Mini/i3) were used. An optical contact angle goniometer (OCA 15 Pro) from DataPhysics Instruments GmbH (Filderstadt, Germany) was used for water contact angle (WCA) measurements, and an A-Series picosecond compact laser micromachining system from Oxford Lasers Ltd. (Oxfordshire, UK) was used to excise the paper substrate as well as prepare both reticles and SETs on the paper substrate. Optical microscope images were obtained with a Keyence VHX 7000 optical microscope (Itasca, IL, USA). Videos S1–S3 were taken with a Vision Research Phantom v2512 high-speed camera (Wayne, NJ, USA) operating at 30,000 frames per second (fps); the Phantom was equipped with a Tokina AT-X PRO MACRO 100 F2.8 D lens. Video playback speeds are shown at 30 fps. For the LMJ-SSP-MS setup, a Sciex 4500 triple quadrupole mass spectrometer (Concord, ON, Canada) was used and operated in the positive ionization mode with a Component Supply Company 304 SS Hypo Tube 30G thin wall electrode along with a T-junction-style LMJ-SSP with a Molex Polymicro fused silica capillary (Part number 1068150026); the carrier liquid was a 94.9:5.0:0.1 MeOH:DI H_2_O:FA volume/volume/volume. Briefly regarding LMJ-SSP-MS [77,78,79], the probe is comprised of two concentric tubes into which a solvent or solvent mixture is pumped; this setup facilitates the formation of a continuously flowing droplet that can be touched to a surface. Through this droplet–surface contact, the surface is sampled, and the analyte:solvent mixture is transported into the ionization source of the MS via the Venturi effect and the pressure drop/vacuum associated with MS [80]. Regarding the LMJ-SSP-MS method, the sampling time was 2.5 s, and multiple reaction monitoring (MRM) in positive ionization mode was used for caffeine, where the mass-to-charge (*m*/*z*) for Q1 was set to 194.9 *m*/*z*, and that for Q3 was set to 138.0 and 110.0 *m*/*z*. Additional MS parameters included a collision gas of 5 pounds per square inch (psi), curtain gas of 20 psi, and ion source temperature of 300 °C. Regarding the FAPA-MS setup, a Prosolia Inc. adjustable mount was used to install and control the FAPA source, and the FAPA source was connected to a Thermo Q Exactive^®^ Orbitrap MS (Bremen, Germany). Briefly, with respect to FAPA-MS, a stream of heated/hot helium (He)/reagent ions is generated by the plasma, and this stream of He/other reagent ions, i.e., the afterglow, enables the desorption/ionization of analyte(s) from a surface into the MS ionization source [81,82,83]. To be compatible with the custom source mount, the DMS was adhered to a hydrophobically-coated glass slide with double-sided tape and placed into the sample holder. The pin-to-capillary FAPA was operated as 30 mA and a helium flow rate of 2.50 L/min. The Q Exactive MS was operated using single-ion monitoring (SIM) in positive ionization mode with *m*/*z* = 195 with the *m*/*z* of protonated caffeine determined to be 195.086. Additional orbitrap parameters included a resolution setting of 140,000 and inlet capillary temperature of 320 °C.

## 3. Results and Discussion

**Figure 1:**
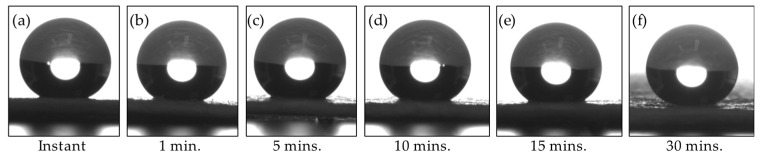
Effect of different coating times on droplet WCA. (**a**) WCA ≈ 145.1° ± 3.3° (*n* = 5), (**b**) WCA ≈ 145.8° ± 2.5° (*n* = 5), (**c**) WCA ≈ 145.7° ± 1.6° (*n* = 5), (**d**) WCA ≈ 145.6° ± 3.6° (*n* = 5), (**e**) WCA ≈ 145.7° ± 1.9° (*n* = 5), and (**f**) WCA ≈ 146.9° ± 3.3° (*n* = 5).

The first step toward improving the DMS substrate involved rendering the filter paper substrate hydrophobic. To democratize this process, an accessible, readily applied coating formulation was chosen for substrate hydrophobization—namely, Aculon^®^. This particular hydrophobization treatment involves a simple two-step coating process, where a base coat, Aculon^®^ AL-A, is applied, which is followed by a topcoat, Aculon^®^ A. In terms of the application process, the paper substrate was wholly submerged into a vial of Aculon^®^ AL-A, and the vial was sealed and sonicated for five minutes, after which the substrate was removed and air-dried for approximately twenty minutes. Once dry, the substrate was wholly submerged into a vial of Aculon^®^ A, sealed, and sonicated for five minutes, after which the substrate was removed and air dried for approximately two minutes. Note the difference in drying times results from Aculon^®^ AL-A being methanol-based and Aculon^®^ A being acetone-based. To better understand the coating process, the paper was submerged in the two different coating solutions without sonication for varying times, as shown in Figure 1. For example, in Figure 1a, the paper substrate was quickly submerged into the vial of Aculon^®^ AL-A solution, instantly removed, and air-dried. The same process was repeated for the substrate using Aculon^®^ A solution.

As Figure 1 shows, the Aculon^®^ coating rapidly renders the substrate hydrophobic, where the WCA achieved by the instantaneous coating (cf. Figure 1a) is nearly the same as the WCA achieved by soaking the substrate in each of the Aculon^®^ solutions for up to 30 min. (cf. Figure 1f). It is important to keep in mind the desired intention for the substrate, i.e., superficial liquid trapping and drying down. Thus, the Aculon^®^ coating must be allowed to sufficiently permeate and coat the porous paper substrate, rather than simply applying a surface-level coating, especially since the final substrate will bear a laser micromachined SET onto which a droplet will be applied, and then confined and dried-down for storage/sampling.

**Figure 2:**
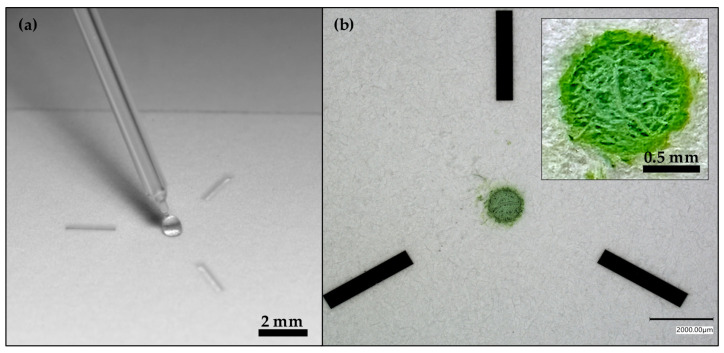
Droplet application process and outcome. (**a**) Photograph of a colorless 1 μL droplet being deposited onto a 1 mm SET flanked by three visual recognition elements. (**b**) photograph of a dried 1 μL droplet that contained green food dye. The inset in (**b**) shows a magnified view of the dried-down droplet on the SET.

To gauge the permeation efficacy of the coating into the filter paper, an SET was laser micromachined onto the coated paper substrate. If the substrate functioned as intended, the liquid should superficially dry-down mostly, if not entirely, within the SET, i.e., maximally resist lateral and transverse diffusion. However, post-laser micromachining, the SET was difficult, if not impossible, to visually discern on the paper substrate to deposit the colored droplet (Figure S1). As such, there became a need to impart visual recognition, or targeting, elements around the SET. Given the attainable precision using the laser micromachining system (i.e., ≈50 μm [75]), a test design for the targeting element was prepared (Figure 2). These different micromachining patterns are easily drafted, modified, and rendered in commercially available software like OnShape and then imported into the laser micromachining software (AlphaCAM 2013 R2 Version 12.5.0.160/Oxford Lasers XY Profiling 291013 AFIT Post Processor/Cimita Version 4.0.21.0). As a note, Whatman Grade 3 (WG3) filter paper was selected over Whatman Grade 1 (WG1) filter paper given that WG3 has greater thickness and smaller pores than WG1 [84], especially since the intention is to minimize liquid flow within the substrate. Relatedly, WG3 was demonstrated to be more amenable to laser micromachining finer design features around an SET relative to WG1 (Figure S2).

Droplet wetting of the different modified paper substrates was observed with a high-speed camera, where there is an observable difference between the droplet wetting Grade 1 (Video S1) versus Grade 3 uncoated filter (Video S2). As well, the droplet being deposited onto the SET was observed (Video S3), where the SET visibly pins the droplet to the surface as a result of increased surface roughness imparted by laser micromachining (Video S4) [75,76,85]. Moreover, the targeting crosshairs create additional permeation entryways for the coating to saturate around the region where the SET was prepared, which further improves the superficiality of liquid drying within the SET boundaries.

With these droplet deposition and wetting demonstrations in place, it was revealed that the hydrophobic coating was indeed functioning, though not maximally, as suggested by the inset in Figure 2b, where the green dye was observed outside of the laser-micromachined boundaries of the SET, which was most likely a result of liquid diffusion and wicking through the paper’s fibers. As an initial next step, the primary laser micromachining parameters that were used to prepare the SETs were assessed—namely, laser power (Video S5) and speed (Video S6). One should keep in mind that a lower laser power will be required to enhance superficiality in terms of surface modification. Specifically, enhanced superficiality means that the SET minimizes, as best as possible, the extent to which the droplet diffuses laterally or transversely. The lower laser power limits increased surface damage and thereby increased potentials for lateral and/or transverse diffusion as well as decreased energy demands on the laser source. In addition, a higher laser writing speed is desirable to decrease the amount of time spent using the laser (i.e., energy and wear-and-tear), as well as time spent preparing the substrate, which could have implications if the process is scaled. Assessments revealed that excessive laser power was detrimental to proper SET functionality; the liquid diffused through the SET and into an underlying paper substrate at 50% laser power. To better characterize the effect of the laser, optical and scanning electron microscopy (SEM) images were obtained for SETs prepared using laser powers of 8, 25, and 50%, as well as a blank (Figures S3 and S4). The optical microscope and SEM images in Figures S3 and S4 show that increasing laser power increasingly damages the substrate. In addition, these images reveal increased material ablation, given that the circular boundary of the SET becomes more visible with increased laser power, along with greater damage near the center of the SET. At 50% laser power, sufficient material is ablated such that the depth resulting from laser micromachining can be observed using the built-in 3D depth composition feature of the Keyence VHX-7000 digital microscope (Figure S5). The SEM images in Figure S4 further reveal that the laser creates microstructuration; the laser ablates material such that the hydrophobic coating is removed and the cellulose fibers are damaged and re-exposed. Since the SETs become wet, i.e., hydrophilicity switch, and retain droplets, these findings confirm that the laser physicochemically changes the surface.

As well, excessive laser writing speed is not ideal for maintaining SET (circular) geometry/uniformity. Overall, the SETs do not maximally retain the liquid within the circular SET boundaries if the laser power and speed are too high. Nonetheless, at this stage in the process, preferred conditions were established as follows: (1) five minutes of Aculon^®^ AL-A coating time with sonication and 20 min. of drying time, (2) five minutes of Aculon^®^ A coating time with sonication and two minutes of drying time, and (3) laser micromachining the SETs with laser parameters set to 8% power (~2.7 milliwatts), 1.50 mm/s speed, and 15 μm pitch. Goniometry, i.e., the measurement of WCAs, was used to assess the substrate at these different preparative stages (Figure S6). Using the refined coating process, the fully coated WG3 filter paper shows a WCA ≈ 138.9° ± 0.8° (*n* = 3) for a 1 µL droplet of DI H_2_O, indicating successful hydrophobization. Then, immediately after depositing a 1 µL droplet of DI H_2_O onto the laser micromachined SET, the droplet shows a WCA ≈ 150.3° ± 1.8° (*n* = 3), indicating that the laser textures the surface and increases the WCA in accordance with the Cassie–Baxter model of wetting, which is especially supported by the laser-induced microstructures presented in Figure S3 [86,87].

**Figure 3:**
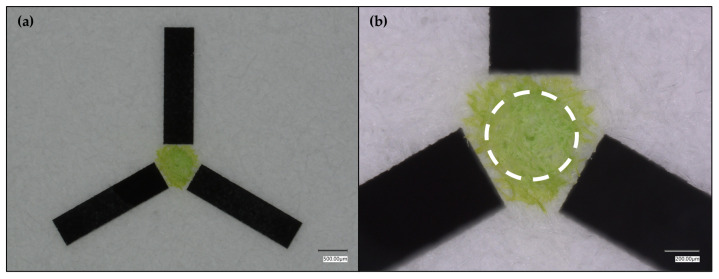
Tightened crosshairs around a green-dyed, 0.55 mm diameter SET. (**a**) shows the full green-dyed SET with the tightened crosshair design. (**b**) shows a magnified view of the SET. The dashed circle in (**b**) approximates the perfect SET boundary.

Given the functional, targeting SET design, we assessed the effect of tightening the crosshairs around the SET to further constrain liquid flow (Figure 3).

**Figure 4:**
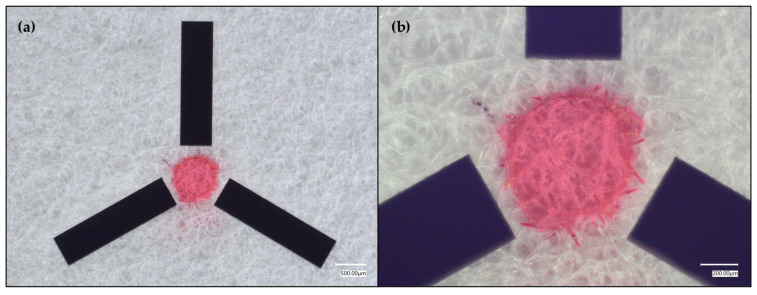
Tightened crosshairs around a pink-dyed, 0.55 mm diameter SET, where the dye was rhodamine 6G. (**a**) shows the full pink-dyed SET with the tightened crosshair design. (**b**) shows a magnified view of the SET.

While the SET nicely confined the liquid within a ca. 0.50 mm diameter region, there was still some liquid diffusion outside of the desired region. This observed diffusion/wicking prompted SET visualization using a different coloring agent rather than the oily green food dye solution that was being used (Figure 4) in case the diffusion/wicking resulted from the visualization agent rather than the laser micromachining method.

**Figure 5:**
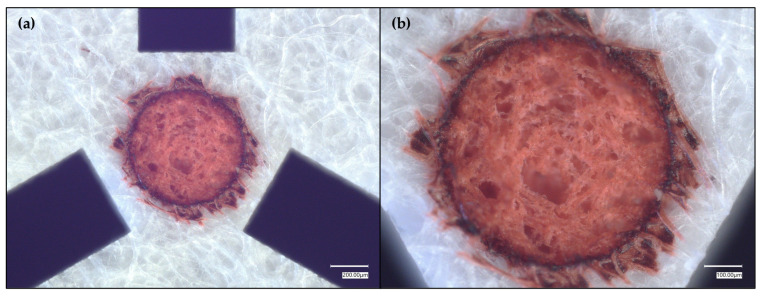
Tightened crosshairs around a red-dyed, 0.55 mm diameter SET, where the dye is Allura Red. (**a**) shows the full red-dyed SET with the tightened crosshair design. (**b**) shows a magnified view of the SET.

As Figure 4 reveals, the SET functioning depends on the relative hydrophobicity of the analyte. While the oiliness of the green food dye solution facilitates some diffusion/wicking, especially owing to the presence of propylene glycol (PG) (logP range ≈ {−1.1, −0.8}) in the food dye [88], there is also some bleeding when using rhodamine 6G (R6G) (logP range ≈ {5.1, 7.2}) as the visualization agent, likely owing to R6G’s amphiphilicty [89], which is an important consideration for future work that might employ such paper-based SETs for liquid sampling. In turn, a relatively more hydrophilic aqueous dye solution, Allura Red (logP range ≈ {−1.3, −0.4}) was tested (Figure 5).

**Figure 6:**
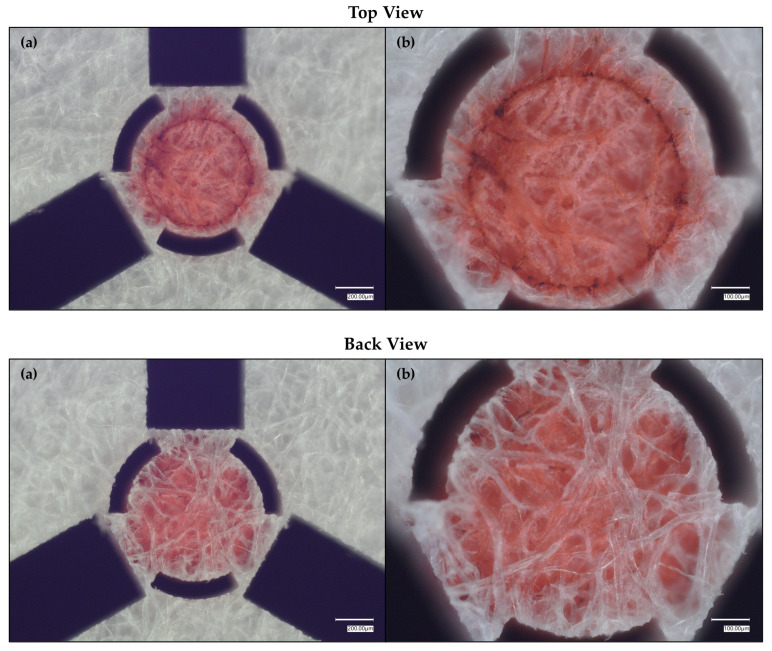
Tightened, arched crosshairs around a red-dyed, 0.55 mm diameter SET, where the dye is Allura Red. The top panel (“Top View”) is the dyed SET’s topside viewed from above using an optical microscope. In the top panel, (**a**) shows the full red-dyed SET with the tightened, arched crosshair design. (**b**) shows a magnified view of the SET. The bottom panel (“Back View”) is the dyed SET’s underside viewed from above using an optical microscope. In the bottom panel, (**a**) shows the back of full red-dyed SET with the tightened, arched crosshair design. (**b**) shows a magnified view of the back of the SET.

As Figure 5 reveals, the Allura red solution performed better with the SET than the R6G solution as well as the green food-dyed solution. Still, there is observable diffusion/wicking outside of the SET. In turn, we once again leveraged the precision of the laser to physically confine liquid flow within the SET by removing additional diffusional/wicking pathways; the modified targeting SET design includes curved arches situated between the targeting crosshairs (Figure 6).

**Figure 7:**
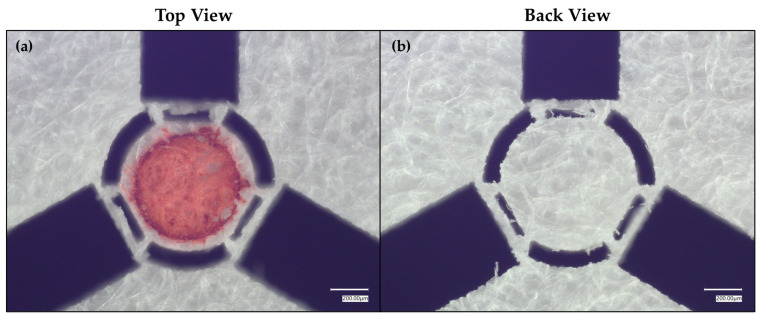
Tightened, arched crosshairs with additional rectangular excisions around a red-dyed, 0.55 mm diameter SET, where the dye is Allura Red. (**a**), i.e., “Top View,” is the dyed SET’s topside viewed from above using an optical microscope, and (**b**), i.e., “Back View,” is the dyed SET’s underside viewed from above using an optical microscope.

While the introduction of arches into the crosshair design improved liquid trapping within the SET, there still remained some excisable area that could be removed to further constrain the liquid within the SET’s boundaries. As such, three additional rectangular regions were removed using the laser micromachining system (Figure 7). To note, as additional areas are removed, the SET will become less firmly adhered; i.e., the structural fidelity is weakened.

As can be seen in Figure 7, the additional rectangular excisions noticeably improve liquid retention within the SET, which is most likely a function of both (1) the reduced paper fibers(fibrils)/pores available for the liquid to diffuse/wick out of the SET and (2) the increased entryways by which the coating can better permeate the space where the SET is laser micromachined (the unspotted SET can be seen in Figure S7). Importantly, there was no observable red coloration on the underside of the SET, indicating that the liquid was confined superficially. To better describe the effect of the improved reticle design on minimizing diffusion outside of the SET, the bleed distance was quantified for each of the three dyes by comparing the diffusion in the initial tightened crosshair design (Figure 3, Figure 4 and Figure 5) to diffusion in the final, refined crosshair design (Figure 7). Specifically, for each of the two designs, using Fiji/ImageJ Version 1.54p, a line was drawn from the center of the SET to the edge of the SET, and this distance was measured. Then, a line was drawn from that same center to each of three exterior points outside of the SET where the particular dye bled the furthest observable distance (Figure S8). The reference distance, which was the radius of the SET, was subtracted from the bleed distance for each, and the average was obtained. As Table 1 shows, the refined crosshair design noticeably improved liquid confinement for all three dyes tested. To further assess the capabilities of the substrate, several additional colorization agents were tested (Figure S9 and Table S1). From these additional tests, it is evident that the oiliness of the solution, rather than the oiliness of the dye compound, has a much greater effect on SET functioning. In particular with the food coloring dyes, the SETs perform well when used with 1 µL of dilute food coloring solution (approximately 1 drop of food dye into ~20 mL of DI H_2_O) versus 1 µL of pure food dye. Therefore, the solute–solvent combination is an important consideration for using the substrate. For this coating–substrate pairing, an aqueous solution performs best.

**Figure 8:**
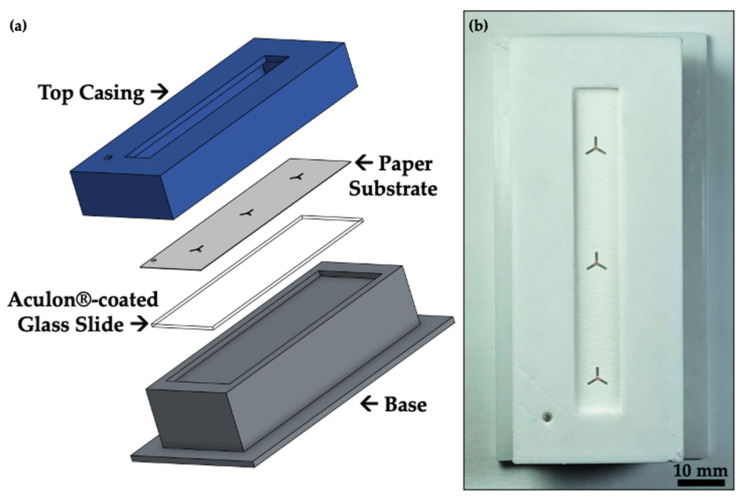
Schematic and demonstration of 3D-printed paper substrate holder for LMJ-SSP-MS sampling. (**a**) shows the sequential placement of the components, which include a 3D-printed base, hydrophobically-coated glass slide to improve flatness as well as prevent the liquid microjunction from contacting the 3D-printed base, the paper substrate, and a top casing to enclose the holder and maintain flatness/rigidity. (**b**) demonstrates the 3D-printed holder with a substrate that has been spotted with Allura Red solution to visualize the SETs in the holder.

With the targeting SETs performing well, we demonstrated the use of the SETs with AIMS, specifically two AIMS techniques: (1) LMJ-SSP-MS (solvent-based extraction/desorption) and (2) FAPA-MS (plasma-based extraction/desorption). For both techniques, the paper substrate should ideally be as flat as possible for uniform sampling; paper is inherently non-uniform. To aid the sampling process for LMJ-SSP-MS, we designed and 3D-printed a sampling holder that keeps the surface flat/level (Figure 8, Figures S10 and S11, and Files S1 and S2).

**Figure 9:**
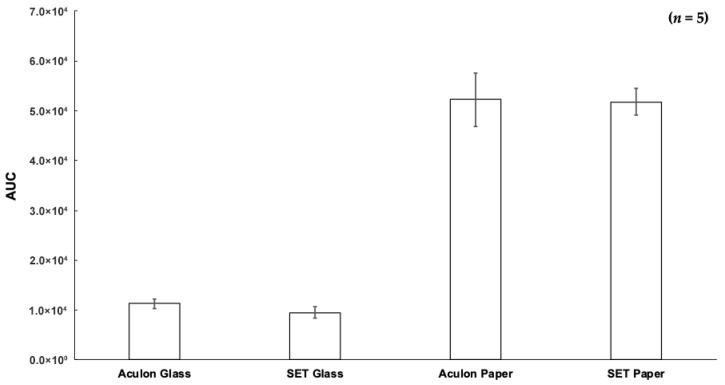
Results of DMS sampling with LMJ-SSP-MS.

Once situated in the holder, 1 μL of 1 ppm caffeine and 100 ppm Allura Red was deposited on the paper substrate. The droplet was allowed to dry under ambient conditions, which took approximately 10 min (Figure S12). The DMSs were then sampled using LMJ-SSP-MS. While one should certainly be mindful about the compatibility between different 3D-printed material(s) and (organic) solvent(s), in this case, because the LMJ-SSP does not directly contact anything besides the paper substrate, 3D-printer filament–solvent compatibility is not a primary concern. In addition, to mitigate the possibility that any solvent from the LMJ-SSP could penetrate the paper substrate and contact the 3D-printed material underneath the paper, a hydrophobic glass slide is placed between the paper substrate and the 3D-printed material. The LMJ-SSP is supported and controlled by a modified 3D printer chassis that enables automated sampling [90]. In turn, the DMSs were sampled, and the substrate/SETs proved successful for surface analysis (Figure 9 and Video S7).

As Figure 9 shows, DMSs prepared on the targeting SETs/modified filter paper yielded an appreciable signal especially since there was no statistically significant difference between the signal acquired from the dried droplet on a targeting SET versus one on the hydrophobic filter paper (i.e., no SET). Note that in Figure 9, “Aculon Glass” represents a hydrophobically-coated glass slide [9% relative standard deviation (RSD)], “SET Glass” represents an SET prepared on hydrophobically-coated glass (12% RSD), “Aculon Paper” represents hydrophobically-coated filter paper (10% RSD), and “SET Paper” represents an SET prepared on hydrophobically-coated filter paper (5% RSD). Note that “AUC” represents “area under the curve.” This finding suggests that the targeting SET is prepared in such a way that there is sufficient surface energy to confine/constrain the droplet while avoiding excessive lateral/transverse diffusion. Figure 9 further illustrates paper as an ideal DMS substrate not only given the sustainability considerations associated with paper as a microfluidic substrate [63,91,92] but also the increased signal intensity that the targeting SETs provided over the glass substrate. Diminished analyte recovery/signal from glass is a known challenge; in this case, the effect is likely due to caffeine’s crystallization on the glass surface [93,94,95,96]. Importantly, though there was comparable performance between the hydrophobically-coated paper with and without targeting SETs, the SETs were crucial to confine/constrain the droplets in defined/known substrate regions, especially if the solution is colorless. Interestingly, this demonstration shows that the surface hydrophobization treatment improved the LMJ-SSP’s ability to desorb analytes from paper (reduced %-RSD) compared to our previous work, where unmodified paper proved to be one of the more challenging substrates to analyze with LMJ-SSP-MS [97]. Finally, this experiment emphasizes the importance of the hydrophobization treatment, the SETs, and the crosshairs for surface sampling. Without the modified SETs, a reliable signal could not be obtained using bare glass or paper as the substrate, especially given the challenges associated with sample spot visualization.

**Figure 10:**
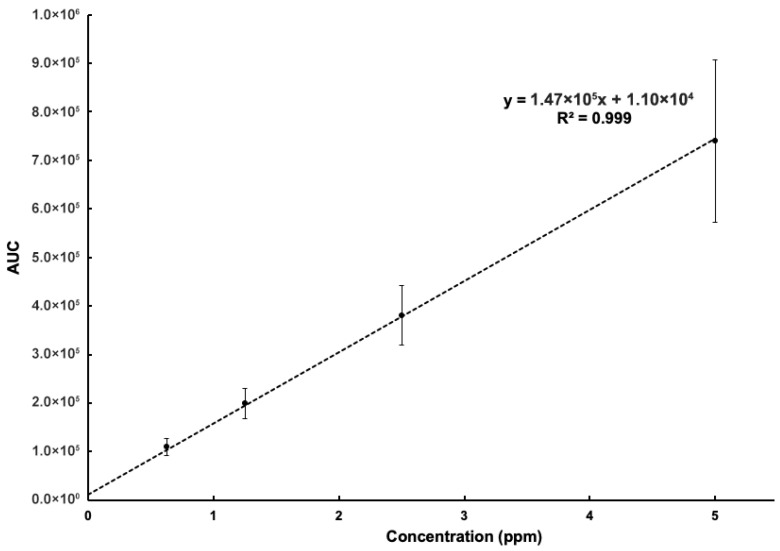
Standard curve prepared using dried-down spots of aqueous caffeine standards (*n* = 5).

A calibration curve was prepared by spotting 1 µL of aqueous caffeine standards ranging from 0.625 to 5 ppm onto the modified SETs, allowing the droplets to dry-down, and then sampling the dried spots using the LMJ-SSP-MS (Figure 10). Using linear regression to obtain the standard error of the intercept from the calibration curve, the limit of detection (LOD) was calculated to be 0.013 ppm (13 ppb), and the limit of quantitation (LOQ) was calculated to be 0.041 ppm (41 ppb). Then, to demonstrate the exhaustiveness of the LMJ-SSP approach, we conducted an experiment wherein the LMJ-SSP was touched to the same DMS for twenty-five sampling instances (Figure S13). As Figure S13 shows, approximately 30.4% of the available caffeine signal from the DMS resulting from 1 µL of 1.25 ppm aqueous caffeine solution is associated with the first sampling instance, followed by 25.4%, then 15.6%, and so on until the signal from the DMS is practically depleted after twenty-five samplings. That is, signal remains after the initial LMJ-SSP sampling of the DMS.

To assess the matrix effect, the average AUC for dried artificial urine (1 µL of 1.25 ppm caffeine in artificial urine solution) was divided by the average AUC for dried aqueous caffeine (1 µL of 1.25 ppm caffeine in DI H_2_O), which represented as a percentage is approximately 51%, indicating that there is a roughly 49% reduction in caffeine signal owing to the artificial urine matrix. As such, sample cleanup (e.g., solid phase microextraction) prior to droplet deposition onto the substrate would likely be required when using this method to prepare and analyze complex matrices like real biofluids.

For comparison, a recent study by Abady, Jeong, and Kwon [98] using HPLC-MS/MS to quantify small molecule cardiovascular drugs in DBSs reports LODs ranging from 0.1 ppb for atorvastatin to 100 ppb for procainamide and LOQs ranging from 0.5 ppb for atorvastatin to 500 ppb for procainamide. In another recent study using dried oral fluid spots [99], Gorziza et al. report the LOQs for several drugs of abuse (e.g., amphetamine, ketamine, etc.) as 0.2 ppb using an LC-MS/MS method. In a final example, Moretti et al. analyze cannabinoids and metabolites in DUSs using LC-MS/MS and report LODs ranging from 0.3 ppb for cannabidiol to 1.9 ppb for 11-hydroxy-delta-9-tetrahydrocannabinol and an LOQ of 10 ppb for the analytes [100]. As such, the LODs/LOQs will vary depending upon the analyte–matrix combination. Overall, our method reported herein shows promise with further refinements, especially since the novel substrate paired with ambient MS does not involve a chromatographic step. Importantly, broader method applicability would require analyte-specific testing and development. For instance, it is known that curcumin binds with cellulosic materials [101,102,103], so one can envision the case where the analyte solution might perform well with the SET, but the analyte could be challenging to remove from the substrate after drying-down using ambient desorption techniques.

**Figure 11:**
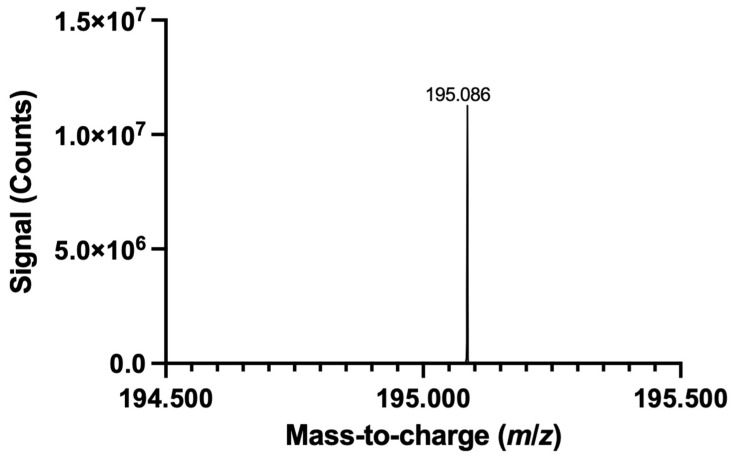
Spectrum resulting from FAPA-MS analysis of DUS containing caffeine.

To complement LMJ-SSP-MS, which is a droplet-based approach, with another AIMS technique, the modified DMS substrate was preliminarily assessed using FAPA-MS, which is a plasma-based approach. In this demonstration, the DMS is placed into the sample holder of the FAPA source mount, and the sample holder containing the DMS is manually steered toward/into the reagent ion stream, at which point sampling begins as the DMS enters the stream; the sampling approach is illustrated in Figure S14. As a proof-of-principle demonstration, a 100 ppm caffeine solution was prepared in artificial urine, and 1 μL of the solution (i.e., 100 nanograms) was spotted onto a targeting SET to prepare a DUS, which was then sampled with FAPA-MS (a full-scan spectrum of the artificial urine is presented in Figure S15). Using SIM, caffeine was successfully detected (Figure 11). To further refine the pairing of the modified paper substrate with FAPA-MS, an internal standard, along with an automated sampling stage and/or means of shuttering the ion stream from the source, could be implemented to minimize sampling variability. For contextualization, surface analysis using FAPA-MS has quantified nicotine from e-liquid samples using thin layer chromatography plates at concentrations of roughly 8840 ppm [104]. In another example, fragrance ingredients like farnesol and patchoulol were qualitatively assessed by FAPA-MS analysis of the liquid sample applied to a glass fiber filter; analyte amounts ranged from 10 to 200 micrograms [105].

## 4. Conclusions

In this paper, we present a laser micromachining-based approach to create a substrate for improved DMS preparation and sampling. Importantly, this hydrophobic substrate uses targeting SETs, or SETs encompassed/flanked by crosshairs/reticles that bolster the coating/hydrophobization process, enhance liquid confinement/trapping within the SET, and aid visual detection/recognition of the SET. Relatedly, we are seeking to leverage the visual detectability of these targeting SETs by combining a camera with a custom object detection-based program, or computer vision, to recognize the crosshairs/reticles for automated, targeted sampling with MS, in support of efforts to further promote the adoption and use of DMS combined with ADI-MS as a (clinically) useful sampling–analysis pairing. Furthermore, by establishing this proof-of-principle work to highlight our fundamental substrate improvement for DMS preparation, future work will seek to leverage these reticle SETs to conduct full (bio)analytical method validation across a range of diverse analytes, (bio)fluids, matrices, and analytical techniques that are both optical and mass spectrometric.

## Figures and Tables

**Table 1:** Assessment of SET bleed distances for three dyes in preliminary versus improved SET design.

Dye	logP Value Range	Initial Average Bleed Distance (µm) (*n* = 3)	Final Average Bleed Distance (µm) (*n* = 3)
Green food dye (PG)	{−1.1, −0.8}	164 ± 28	50 ± 13
R6G	{5.1, 7.2}	121 ± 36	77 ± 8
Allura Red AC	{−1.3, −0.4}	120 ± 5	28 ± 8

## Data Availability

The original contributions presented in this paper are included in the article/Supplementary Material. Further inquiries can be directed to the corresponding author.

## Supplementary Materials

The following supporting information can be downloaded at https://www.mdpi.com/article/10.3390/mi17050559/s1, Table S1: Summary of visualization agent estimated logP value ranges and bleed distances; Figure S1: Droplets at various dry-down stages on surface energy traps (SETs) prepared on laser micromachined Whatman Grade 3 (WG3) filter paper; Figure S2: SETs prepared and visualized on Whatman Grade 1 filter paper; Figure S3: Optical microscopy images of WG3 filter paper bearing SETs prepared at different laser powers; Figure S4: Scanning electron microscopy images of WG3 filter paper bearing SETs prepared at different laser powers; Figure S5: Three-dimensional (3D) depth composition of an SET at 50% laser power; Figure S6: WG3 filter paper at various stages throughout the coating process; Figure S7: Blank/unspotted targeting SET design with three curved arches and three rectangular excisions; Figure S8: Example showing how the reference line (Solid) was drawn from the center to the edge of the SET and the three “bleed” distance measurement lines (Dashed) were drawn from the center of the SET to three exterior bleeding edges; Figure S9: Different visualization agents on 0.55 mm SETs; Figure S10: (a) Length/width dimensions of the holder base in millimeters (mm). (b) The heights/thicknesses for the various aspects of the holder base; Figure S11: (a) Length/width dimensions of the holder clamp/top in millimeters (mm). (b) The heights/thicknesses for the various aspects of the holder clamp/top; Figure S12: Time lapse of 1 µL deionized water droplet drying-down on paper substrate under ambient conditions; Figure S13: Chromatogram showing caffeine signal depletion of a DMS after repeated sampling by the liquid microjunction—surface sampling probe (LMJ-SSP); Figure S14: Demonstration of the flowing atmospheric pressure afterglow (FAPA) sampling approach; Figure S15: Full-scan spectrum resulting from FAPA-MS analysis of 1 µL of undiluted Pickering Laboratories artificial urine spotted onto targeting SET; Video S1: Droplet wetting WG1 filter paper; Video S2: Droplet wetting WG3 filter paper; Video S3: Droplet wetting targeting SET; Video S4: Targeting SET trapping droplet; Video S5: Laser power-varied target SETs; Video S6: Laser-speed varied targeting SETs; Video S7: LMJ-SSP-MS demonstration; File S1: DMS holder base; File S2: DMS holder clamp.

## Abbreviations

The following abbreviations are used in this manuscript:

μL	Microliter(s)
μm	Micrometer(s)
AIMS	Ambient ionization mass spectrometry
DBS	Dried blood spot(s)
DI	Deionized
DMS	Dried matrix spot
DS(a)S	Dried saliva spot
D(S)S	Dried (sample) spot
DUS	Dried urine spot
FA	Formic acid
FAPA	Flowing atmospheric pressure afterglow
(HP)LC	(High-performance) liquid chromatography
LMJ-SSP	Liquid microjunction–surface sampling probe
LOD(s)	Limit(s) of detection
LOQ(s)	Limit(s) of quantitation
MeOH	Methanol
mm	Millimeter(s)
MRM	Multiple reaction monitoring
MS	Mass spectrometry
OCA	Optical contact angle
PETG	Polyethylene glycol
PG	Propylene glycol
ppm	Part(s) per million
R6G	Rhodamine 6G
RSD	Relative standard deviation
SET(s)	Surface energy trap(s)
SIM	Single ion monitoring
WCA	Water contact angle
WG1	Whatman Grade 1
WG3	Whatman Grade 3

## References

[1] Abu-Rabie P., Spooner N. (2011). Dried Matrix Spot Direct Analysis: Evaluating the Robustness of a Direct Elution Technique for Use in Quantitative Bioanalysis. Bioanalysis.

[2] Déglon J., Leuthold L.A., Thomas A. (2015). Potential Missing Steps for a Wide Use of Dried Matrix Spots in Biomedical Analysis. Bioanalysis.

[3] Fabris A.L., Yonamine M. (2021). Dried Matrix Spots in Forensic Toxicology. Bioanalysis.

[4] Zimmer J.S., Christianson C.D., Johnson C.J., Needham S.R. (2013). Recent Advances in the Bioanalytical Applications of Dried Matrix Spotting for the Analysis of Drugs and Their Metabolites. Bioanalysis.

[5] Miller T.C., Havrilla G.J. (2004). Nanodroplets: A New Method for Dried Spot Preparation and Analysis. X-Ray Spectrom..

[6] Perez J.W., Pantazides B.G., Watson C.M., Thomas J.D., Blake T.A., Johnson R.C. (2015). Enhanced Stability of Blood Matrices Using a Dried Sample Spot Assay To Measure Human Butyrylcholinesterase Activity and Nerve Agent Adducts. Anal. Chem..

[7] Rago B., Liu J., Tan B., Holliman C. (2011). Application of the Dried Spot Sampling Technique for Rat Cerebrospinal Fluid Sample Collection and Analysis. J. Pharm. Biomed. Anal..

[8] Kummari S., Panicker L.R., Rao Bommi J., Karingula S., Sunil Kumar V., Mahato K., Goud K.Y. (2023). Trends in Paper-Based Sensing Devices for Clinical and Environmental Monitoring. Biosensors.

[9] Pulivarthi D., Chovatiya J., Jagani R., Andra S.S. (2025). Dried Matrix Spots: An Underutilized and Unexplored Technology in India. Chem. Res. Toxicol..

[10] Colozza N., Caratelli V., Moscone D., Arduini F. (2021). Paper-Based Devices as New Smart Analytical Tools for Sustainable Detection of Environmental Pollutants. Case Stud. Chem. Environ. Eng..

[11] Niu Z., Shi J., Xu Z., Zheng Y., Xiang Z., Zhao J., Zhang Z. (2021). Paper-Based Filter Membrane for High-Efficient Sampling and Direct Mass Spectrometric Analysis of Siloxanes in Outdoor Air. Atmos. Environ..

[12] Meredith N.A., Quinn C., Cate D.M., Reilly T.H., Volckens J., Henry C.S. (2016). Paper-Based Analytical Devices for Environmental Analysis. Analyst.

[13] Jacques A.L.B., Santos M.K., Gorziza R.P., Limberger R.P. (2022). Dried Matrix Spots: An Evolving Trend in the Toxicological Field. Forensic Sci. Med. Pathol..

[14] Richert W., Korzeniewski K. (2024). The Use of Dried Matrix Spots as an Alternative Sampling Technique for Monitoring Neglected Tropical Diseases. Pathogens.

[15] Hernandes V.V., Zeyda M., Wisgrill L., Warth B. (2025). Dried Blood Spots Analysis for Targeted and Non-Targeted Exposomics. Environ. Int..

[16] Heikenfeld J., Jajack A., Feldman B., Granger S.W., Gaitonde S., Begtrup G., Katchman B.A. (2019). Accessing Analytes in Biofluids for Peripheral Biochemical Monitoring. Nat. Biotechnol..

[17] Fischer S., Obrist R., Ehlert U. (2019). How and When to Use Dried Blood Spots in Psychoneuroendocrinological Research. Psychoneuroendocrinology.

[18] Frederick D.L. (2012). Toxicology Testing in Alternative Specimen Matrices. Clin. Lab. Med..

[19] Cheng J.Y., Hui J.W., Chan W., So M., Hong Y., Leung W., Ku K., Yeung H., Lo K., Fung K. (2023). Interpol Review of Toxicology 2019–2022. Forensic Sci. Int. Synerg..

[20] Kiseleva O.I., Ikhalaynen Y.A., Kurbatov I.Y., Arzumanian V.A., Kryukova P.A., Poverennaya E.V. (2025). Dried Spot Paradigm: Problems and Prospects in Proteomics. Int. J. Mol. Sci..

[21] Abdel-Rehim A., Abdel-Rehim M. (2014). Dried Saliva Spot as a Sampling Technique for Saliva Samples. Biomed. Chromatogr..

[22] Han Y., Li X.-L., Zhang M., Wang J., Zeng S., Min J.Z. (2022). Potential Use of a Dried Saliva Spot (DSS) in Therapeutic Drug Monitoring and Disease Diagnosis. J. Pharm. Anal..

[23] Numako M., Takayama T., Noge I., Kitagawa Y., Todoroki K., Mizuno H., Min J.Z., Toyo’oka T. (2016). Dried Saliva Spot (DSS) as a Convenient and Reliable Sampling for Bioanalysis: An Application for the Diagnosis of Diabetes Mellitus. Anal. Chem..

[24] Dvořák M., Maršala R., Kubáň P. (2023). In-Vial Dried Urine Spot Collection and Processing for Quantitative Analyses. Anal. Chim. Acta.

[25] Schmidt J., Lindemann V., Olsen M., Cramer B., Humpf H.-U. (2021). Dried Urine Spots as Sampling Technique for Multi-Mycotoxin Analysis in Human Urine. Mycotoxin Res..

[26] Suwanvecho C., Vyleťalová L., Přívratská N., Laolertworakul P., Turoňová D., Vošmik M., Krčmová L.K., Svec F. (2025). Dried Urine Spot as a Stable, Green, and Practical Microsampling Tool in Clinical Practice for Quantification of Neopterin and Creatinine. Green Chem..

[27] Clarke E.G.C., Sowter S.A. (1964). Continuous Flow Spotting for Paper Chromatograms. Nature.

[28] Abu-Rabie P., Denniff P., Spooner N., Brynjolffssen J., Galluzzo P., Sanders G. (2011). Method of Applying Internal Standard to Dried Matrix Spot Samples for Use in Quantitative Bioanalysis. Anal. Chem..

[29] Aramendía M., Rello L., Vanhaecke F., Resano M. (2012). Direct Trace-Elemental Analysis of Urine Samples by Laser Ablation-Inductively Coupled Plasma Mass Spectrometry after Sample Deposition on Clinical Filter Papers. Anal. Chem..

[30] Vuckovic D., Issaq H.J., Veenstra T.D. (2013). Chapter 4—Sample Preparation in Global Metabolomics of Biological Fluids and Tissues. Proteomic and Metabolomic Approaches to Biomarker Discovery.

[31] Jing J., Liu B., Zhao J., Chen P., Xu X. (2025). Modified Fully Automated Dried Blood Spot Sample Preparation System and Its Application in Steroid Ester Detection. Anal. Chem..

[32] Lange T., Thomas A., Walpurgis K., Thevis M. (2020). Fully Automated Dried Blood Spot Sample Preparation Enables the Detection of Lower Molecular Mass Peptide and Non-Peptide Doping Agents by Means of LC-HRMS. Anal. Bioanal. Chem..

[33] Resano M., Belarra M.A., García-Ruiz E., Aramendía M., Rello L. (2018). Dried Matrix Spots and Clinical Elemental Analysis. Current Status, Difficulties, and Opportunities. TrAC Trends Anal. Chem..

[34] Hayeems R.Z., Miller F.A., Carroll J.C., Little J., Allanson J., Bytautas J.P., Chakraborty P., Wilson B.J. (2013). Primary Care Role in Expanded Newborn Screening: After the Heel Prick Test. Can. Fam. Physician.

[35] Overgaard C., Knudsen A. (1999). Pain-Relieving Effect of Sucrose in Newborns during Heel Prick. Biol. Neonate.

[36] Jing J., Shan Y., Liu Z., Yan H., Xiang P., Chen P., Xu X. (2022). Automated Online Dried Blood Spot Sample Preparation and Detection of Anabolic Steroid Esters for Sports Drug Testing. Drug Test. Anal..

[37] Garzinsky A., Thomas A., Guddat S., Görgens C., Dib J., Thevis M. (2023). Dried Blood Spots for Doping Controls—Development of a Comprehensive Initial Testing Procedure with Fully Automated Sample Preparation. Biomed. Chromatogr..

[38] Choudhury A.M., Sarikonda H., Khan I.I., Deol J., Tirmizi Z. (2024). Liquid Handling Technologies: A Study through Major Discoveries and Advancements. Adv. Int. J. Multidiscip. Res..

[39] Kong F., Yuan L., Zheng Y.F., Chen W. (2012). Automatic Liquid Handling for Life Science: A Critical Review of the Current State of the Art. J. Lab. Autom..

[40] Eppendorf (2017). Master Your Challenging Liquids. https://www.eppendorf.com/product-media/doc/en/336668/Liquid-Handling_Poster_Pipette_Multipette_Master-Your-Challenging-Liquids.pdf.

[41] Mettler Toledo Pipette Challenging Liquids. Know How. https://www.mt.com/us/en/home/library/know-how/rainin-pipettes/pipette-challenging-liquids.html.

[42] Söderholm S., Artimo P. (2021). Techniques for Pipetting Challenging Liquids. https://www.sartorius.com/resource/blob/1085646/d4bf11ef41cbbc9c7ebdcc88ecea4846/how-to-pipette-challenging-liquids-application-guide-en-l-sa-1--data.pdf.

[43] Delaby C., Gabelle A., Meynier P., Loubiere V., Vialaret J., Tiers L., Ducos J., Hirtz C., Lehmann S. (2014). Development and Validation of Dried Matrix Spot Sampling for the Quantitative Determination of Amyloid β Peptides in Cerebrospinal Fluid. Clin. Chem. Lab. Med..

[44] Skogvold H.B., Rootwelt H., Reubsaet L., Elgstøen K.B.P., Wilson S.R. (2023). Dried Blood Spot Analysis with Liquid Chromatography and Mass Spectrometry: Trends in Clinical Chemistry. J. Sep. Sci..

[45] Carvalho J., Rosado T., Barroso M., Gallardo E. (2019). Determination of Antiepileptic Drugs Using Dried Saliva Spots. J. Anal. Toxicol..

[46] Chantada-Vázquez M.P., Moreda–Piñeiro J., Cantarero–Roldán A., Bermejo-Barrera P., Moreda-Piñeiro A. (2018). Development of Dried Serum Spot Sampling Techniques for the Assessment of Trace Elements in Serum Samples by LA-ICP-MS. Talanta.

[47] Li W., Tse F.L.S. (2010). Dried Blood Spot Sampling in Combination with LC-MS/MS for Quantitative Analysis of Small Molecules. Biomed. Chromatogr..

[48] Qi Z., Zhang S., Li J., Qian S., Song D., Ye X. (2025). LC-MS/MS Analysis of Five Antibiotics in Dried Blood Spots for Therapeutic Drug Monitoring of ICU Patients. J. Chromatogr. B.

[49] Sathyanarayanan A., Mysore Somashekara D. (2022). Dried Spot Sample and Its Drug Detection Using LC-MS/MS: Trends and Advances in Matrix Collection and Bioanalytics. J. Appl. Pharm. Sci..

[50] Maritz L., Woudberg N.J., Bennett A.C., Soares A., Lapierre F., Devine J., Kimberg M., Bouic P.J. (2021). Validation of High-Throughput, Semiquantitative Solid-Phase SARS Coronavirus-2 Serology Assays in Serum and Dried Blood Spot Matrices. Bioanalysis.

[51] Roberts J.L., Ryan M.J., Whiley L., Gay M., Nambiar V., Holmes E., Nicholson J.K., Wist J., Gray N., Lawler N.G. (2025). Dried Blood Spot Microsampling: A Semi-Quantitative 4D-Lipidomics Approach Using Ultrahigh-Performance Liquid Chromatography—High-Resolution Mass Spectrometry (UHPLC—HRMS). Talanta.

[52] Chen S., Wan Q., Badu-Tawiah A.K. (2016). Mass Spectrometry for Paper-Based Immunoassays: Toward On-Demand Diagnosis. J. Am. Chem. Soc..

[53] Pizzi E., Halvorsen T.G., Koehler C.J., Reubsaet L. (2021). Next-Generation Dried Blood Spot Samplers for Protein Analysis: Describing Trypsin-Modified Smart Sampling Paper. Separations.

[54] Spooner N. (2013). A Dried Blood Spot Update: Still an Important Bioanalytical Technique?. Bioanalysis.

[55] Zhang Z., Xu W., Manicke N.E., Cooks R.G., Ouyang Z. (2012). Silica Coated Paper Substrate for Paper-Spray Analysis of Therapeutic Drugs in Dried Blood Spots. Anal. Chem..

[56] Moretti M., Manfredi A., Freni F., Previderé C., Osculati A.M.M., Grignani P., Tronconi L., Carelli C., Vignali C., Morini L. (2021). A Comparison between Two Different Dried Blood Substrates in Determination of Psychoactive Substances in Postmortem Samples. Forensic Toxicol..

[57] Zakaria R., Allen K.J., Koplin J.J., Roche P., Greaves R.F. (2016). Advantages and Challenges of Dried Blood Spot Analysis by Mass Spectrometry across the Total Testing Process. EJIFCC.

[58] Chaudhury K., Kar S., Chakraborty S. (2016). Diffusive Dynamics on Paper Matrix. Appl. Phys. Lett..

[59] Kumar A., Hatayama J., Soucy A., Carpio E., Rahmani N., Anagnostopoulos C., Faghri M. (2024). Fluid Flow Dynamics in Partially Saturated Paper. Micromachines.

[60] MacDonald B.D. (2018). Flow of Liquids through Paper. J. Fluid Mech..

[61] Patari S., Mahapatra P.S. (2020). Liquid Wicking in a Paper Strip: An Experimental and Numerical Study. ACS Omega.

[62] Ray P., Agrawal P., Kumar P. (2025). Manipulating Fluid Flow Behavior in Microporous Paper to Achieve Better Mixing in Lateral Flow Devices. Phys. Fluids.

[63] Aryal P., Henry C.S. (2024). Advancements and Challenges in Microfluidic Paper-Based Analytical Devices: Design, Manufacturing, Sustainability, and Field Applications. Front. Lab Chip Technol..

[64] Dudnyk Y., Kulha P., Procházka V., Nyström G., Geiger T. (2025). Printed Circuit Board Substrates Derived from Lignocellulose Nanofibrils for Sustainable Electronics Applications. Sci. Rep..

[65] Norambuena J., Araya-Hermosilla R., Sandoval S.S., Crisóstomo S., Martínez J., Silva N. (2025). Cellulose Paper as a Sustainable Substrate for the Bacterial Biosynthesis of Metal Nanoparticles. Int. J. Biol. Macromol..

[66] Oloyede O.O., Lignou S. (2021). Sustainable Paper-Based Packaging: A Consumer’s Perspective. Foods.

[67] Garza K., Clarke W. (2022). Dried Blood Spots and Beyond.

[68] Thangavelu M.U., Wouters B., Kindt A., Reiss I.K.M., Hankemeier T. (2023). Blood Microsampling Technologies: Innovations and Applications in 2022. Anal. Sci. Adv..

[69] Biagini D., Antoni S., Ghimenti S., Bonini A., Vivaldi F., Angelucci C., Riparbelli C., Cuttano A., Fuoco R., Di Francesco F. (2022). Methodological Aspects of Dried Blood Spot Sampling for the Determination of Isoprostanoids and Prostanoids. Microchem. J..

[70] Lim J., Hwang J., Min H., Wester M., Kim C., Valera E., Kong H.J., Bashir R. (2024). Dried Blood Matrix as a New Material for the Detection of DNA Viruses. Adv. Healthc. Mater..

[71] Rosendo L.M., Gonçalves R., Martins R., Castro V., Rosado T., Barroso M., Gallardo E. (2025). Dried Matrix Spots for the Determination of Opiates and Opioids: Methodological Advances and Applications. Molecules.

[72] Baillargeon K.R., Brooks J.C., Miljanic P.R., Mace C.R. (2022). Patterned Dried Blood Spot Cards for the Improved Sampling of Whole Blood. ACS Meas. Sci. Au.

[73] Kim J.-H., Woenker T., Adamec J., Regnier F.E. (2013). Simple, Miniaturized Blood Plasma Extraction Method. Anal. Chem..

[74] Hassan M., Reddy D.O., Alashraf A.R., Brown S., Oleschuk R.D. (2025). Microarray-Based Nanoextraction Combined with Ambient Ionization Mass Spectrometry for Analysis of Drugs of Abuse in Wastewater. Anal. Chem..

[75] Zhang L., Salomons T.T., Reddy D., Hillen P., Oleschuk R. (2023). A Universally Adaptable Micropatterning Method through Laser-Induced Wettability Inversion. Sens. Actuators B Chem..

[76] Zhang L., Reddy D.O., Salomons T.T., Oleschuk R.D. (2024). Micro “Hyper-Channels” on Laser-Refined Cellulose Structures. Small Methods.

[77] Van Berkel G.J., Sanchez A.D., Quirke J.M.E. (2002). Thin-Layer Chromatography and Electrospray Mass Spectrometry Coupled Using a Surface Sampling Probe. Anal. Chem..

[78] Van Berkel G.J., Kertesz V., Koeplinger K.A., Vavrek M., Kong A.-N.T. (2008). Liquid Microjunction Surface Sampling Probe Electrospray Mass Spectrometry for Detection of Drugs and Metabolites in Thin Tissue Sections. J. Mass. Spectrom..

[79] Van Berkel G.J., Kertesz V., King R.C. (2009). High-Throughput Mode Liquid Microjunction Surface Sampling Probe. Anal. Chem..

[80] Simon D., Oleschuk R. (2021). The Liquid Micro Junction-Surface Sampling Probe (LMJ-SSP); a Versatile Ambient Mass Spectrometry Interface. Analyst.

[81] Aghaei M., Bogaerts A. (2021). Flowing Atmospheric Pressure Afterglow for Ambient Ionization: Reaction Pathways Revealed by Modeling. Anal. Chem..

[82] Shelley J.T., Chan G.C.-Y., Hieftje G.M. (2012). Understanding the Flowing Atmospheric-Pressure Afterglow (FAPA) Ambient Ionization Source through Optical Means. J. Am. Soc. Mass. Spectrom..

[83] Shelley J.T., Badal S.P., Engelhard C., Hayen H. (2018). Ambient Desorption/Ionization Mass Spectrometry: Evolution from Rapid Qualitative Screening to Accurate Quantification Tool. Anal. Bioanal. Chem..

[84] Cytiva (2024). Whatman Filter Paper Grades Guide.

[85] Reddy D.O., Zhang L., Covey T.R., Oleschuk R.D. (2025). Design and Preparation of a Simplified Microdroplet Generation Device for Nanoliter Volume Collection and Measurement with Liquid Microjunction–Surface Sampling Probe–Mass Spectrometry. Droplet.

[86] Cassie A.B.D., Baxter S. (1944). Wettability of Porous Surfaces. Trans. Faraday Soc..

[87] Deng Y., Peng C., Dai M., Lin D., Ali I., Alhewairini S.S., Zheng X., Chen G., Li J., Naz I. (2020). Recent Development of Super-Wettable Materials and Their Applications in Oil-Water Separation. J. Clean. Prod..

[88] Club House Green Food Colouring. https://www.clubhouse.ca/en-ca/products/baking/food-colours/green-food-colour.

[89] MilliporeSigma Rhodamine 6G Perchlorate. https://www.sigmaaldrich.com/CA/en/product/aldrich/252441?srsltid=AfmBOopXR9PW5L2M_-TwATFeluKh5RC2ai8Tk6N_7Ho5sLUKIeT6OfGn.

[90] Hermann M., Metwally H., Yu J., Smith R., Tomm H., Kaufmann M., Ren K.Y.M., Liu C., LeBlanc Y., Covey T.R. (2023). 3D Printer Platform and Conductance Feedback Loop for Automated Imaging of Uneven Surfaces by Liquid Microjunction-surface Sampling Probe Mass Spectrometry. Rapid Commun. Mass Spectrom..

[91] Brito-Pereira R., Macedo A.S., Ribeiro C., Cardoso V.F., Lanceros-Méndez S. (2022). Natural Based Reusable Materials for Microfluidic Substrates: The Silk Road towards Sustainable Portable Analytical Systems. Appl. Mater. Today.

[92] Ongaro A.E., Ndlovu Z., Sollier E., Otieno C., Ondoa P., Street A., Kersaudy-Kerhoas M. (2022). Engineering a Sustainable Future for Point-of-Care Diagnostics and Single-Use Microfluidic Devices. Lab Chip.

[93] Manicke N.E., Wiseman J.M., Ifa D.R., Cooks R.G. (2008). Desorption Electrospray Ionization (DESI) Mass Spectrometry and Tandem Mass Spectrometry (MS/MS) of Phospholipids and Sphingolipids: Ionization, Adduct Formation, and Fragmentation. J. Am. Soc. Mass. Spectrom..

[94] Ifa D.R., Wu C., Ouyang Z., Cooks R.G. (2010). Desorption Electrospray Ionization and Other Ambient Ionization Methods: Current Progress and Preview. Analyst.

[95] Sarfraz A., Simo A., Fenger R., Christen W., Rademann K., Panne U., Emmerling F. (2012). Morphological Diversity of Caffeine on Surfaces: Needles and Hexagons. Cryst. Growth Des..

[96] Wiseman J.M., Ifa D.R., Zhu Y., Kissinger C.B., Manicke N.E., Kissinger P.T., Cooks R.G. (2008). Desorption Electrospray Ionization Mass Spectrometry: Imaging Drugs and Metabolites in Tissues. Proc. Natl. Acad. Sci. USA.

[97] Metwally H., Agrawal P., Smith R., Liu C., LeBlanc Y., Covey T.R., Oleschuk R. (2020). Detection of Opioids on Mail/Packages Using Open Port Interface Mass Spectrometry (OPI-MS). J. Am. Soc. Mass Spectrom..

[98] Abady M.M., Jeong J.-S., Kwon H.-J. (2024). Dried Blood Spot Sampling Coupled with Liquid Chromatography-Tandem Mass for Simultaneous Quantitative Analysis of Multiple Cardiovascular Drugs. J. Chromatogr. B.

[99] Gorziza R., Cox J., Pereira Limberger R., Arroyo-Mora L.E. (2020). Extraction of Dried Oral Fluid Spots (DOFS) for the Identification of Drugs of Abuse Using Liquid Chromatography Tandem Mass Spectrometry (LC-MS/MS). Forensic Chem..

[100] Moretti M., Freni F., Carelli C., Previderé C., Grignani P., Vignali C., Cobo-Golpe M., Morini L. (2021). Analysis of Cannabinoids and Metabolites in Dried Urine Spots (DUS). Molecules.

[101] Casanova F., Pereira C.F., Ribeiro A.B., Castro P.M., Freixo R., Martins E., Tavares-Valente D., Fernandes J.C., Pintado M.E., Ramos Ó.L. (2023). Biological Potential and Bioaccessibility of Encapsulated Curcumin into Cetyltrimethylammonium Bromide Modified Cellulose Nanocrystals. Pharmaceuticals.

[102] Tian Y., Jia D., Dirican M., Cui M., Fang D., Yan C., Xie J., Liu Y., Li C., Fu J. (2022). Highly Soluble and Stable, High Release Rate Nanocellulose Codrug Delivery System of Curcumin and AuNPs for Dual Chemo-Photothermal Therapy. Biomacromolecules.

[103] Zubair M., Husain F.M., Siddiqui Z.H., Khan Athar A., Alamri M., Faisal Muhammad Ali Albudair S. (2026). Synthesis and Characterization of Curcumin-Loaded Cellulose Nanoparticles Targeting Bacterial Quorum Sensing and Biofilms in Foodborne Bacteria. Front. Microbiol..

[104] Heide M., Engelhard C. (2023). Chemical Analysis of Electronic Cigarette Liquids (e-Liquids) and Direct Nicotine Quantitation Using Surface-Assisted Flowing Atmospheric-Pressure Afterglow Desorption/Ionization Mass Spectrometry (SA-FAPA-MS). RSC Adv..

[105] Guć M., Cegłowski M., Pawlaczyk M., Kurczewska J., Reszke E., Schroeder G. (2021). Application of FAPA Mass Spectrometry for Analysis of Fragrance Ingredients Used in Cosmetics. Measurement.

