# Supplementary material for: Spot on: A Laser Micromachining-Based Approach to Improve Dried Matrix Spot Preparation with Proof-of-Principle Analytical Demonstrations Using Ambient Ionization Mass Spectrometry
Source: Micromachines (Basel). 2026 Apr 30;17(5):559. doi: 10.3390/mi17050559 (PMC13209217; doi:10.3390/mi17050559)
Supplement: Supplementary file 1 [file micromachines-17-00559-s001.zip › Supporting Information.pdf]

Supporting Information

Spot on: A Laser Micromachining-Based Approach to Improve Dried Matrix Spot Preparation with Proof-of-Principle Analytical Demonstrations Using Ambient Ionization Mass Spectrometry

Daniel O. Reddy,[1] Malek Hassan,[1] Jonathan O. Graham,[2] Jared Viggers,[2] Katherine E. Williams,[3] Randy E. Ellis,[3] Thomas R. Covey,[4] Jacob T. Shelley,[2] and Richard D. Oleschuk[1]*

[1]Department of Chemistry, Queen's University, Kingston, ON K7L 3N6, Canada

[2]Department of Chemistry and Chemical Biology, Rensselaer Polytechnic Institute, Troy, NY 12180, USA

[3]School of Computing, Queen's University, Kingston, ON K7L 2N8, Canada

[4]SCIEX, Concord, ON L4K 4V8, Canada

[*]Correspondence: oleschuk@queensu.ca

Table S1. Summary of visualization agent estimated logP value ranges and bleed distances.

**Visualization Agent**	**logP Value Range**	**Average Bleed Distance (µm) (*n* = 3)**
Dilute yellow food dye (likely tartrazine) (contains PG)	{-10.2, -1.6}	73 ± 10
Undiluted yellow food dye (likely tartrazine) (contains PG)	{-10.2, -1.6}	142 ± 4
Dilute red food dye (likely Allura Red AC) (contains PG)	{-1.3, -0.4}	83 ± 9
Undiluted red food dye (likely Allura Red AC) (contains PG)	{-1.3, -0.4}	436 ± 19
Propylene glycol (PG)	{-1.1, -0.8}	N/A
Dilute Nile Blue	{1.1, 2.9}	89 ± 8
Dilute Indigo	{2.7, 3.7}	89 ± 19
Dilute blue food dye (likely Brilliant Blue FCF) (contains PG)	{8.3, 9.0}	85 ± 11
Undiluted blue food dye (likely Brilliant Blue FCF) (contains PG)	{8.3, 9.0}	111 ± 5

Note that PG represents “propylene glycol.”

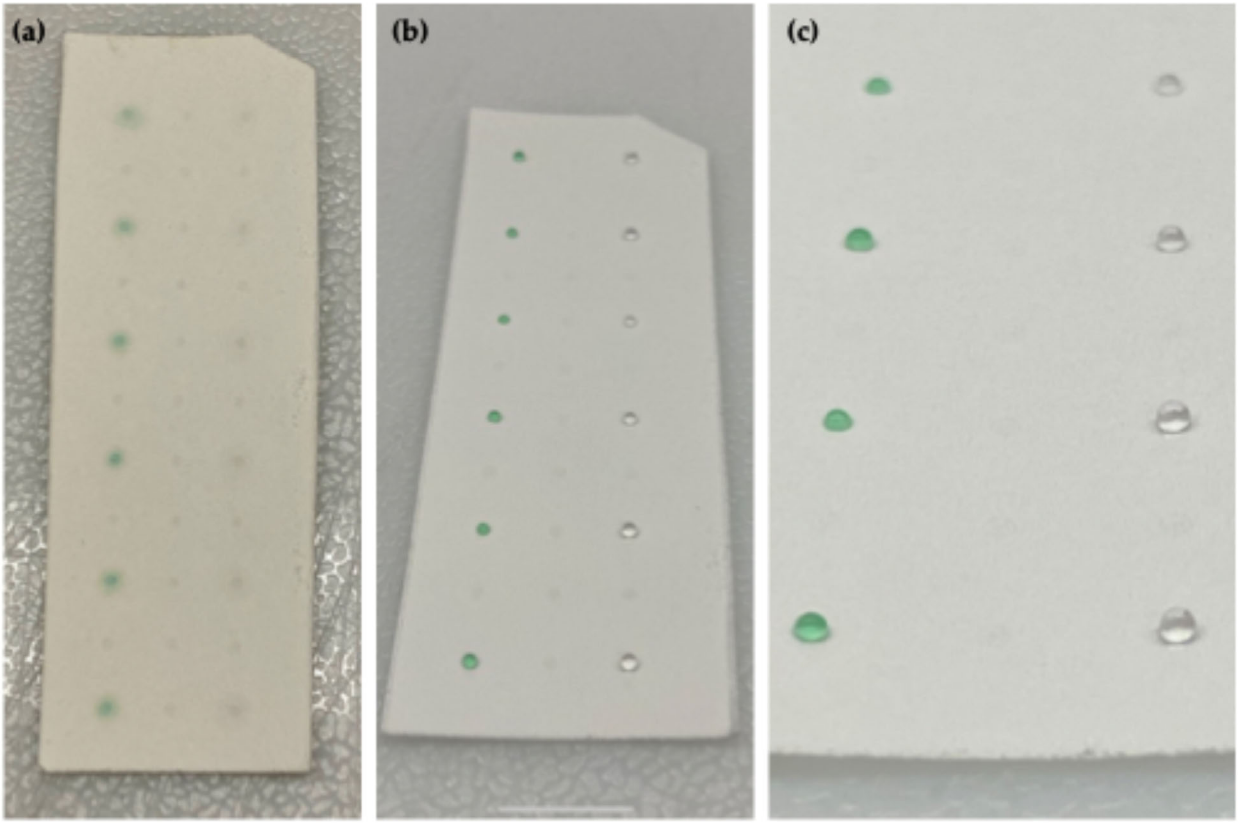

Figure S1. Droplets at various dry-down stages on surface energy traps (SETs) prepared on laser micromachined Whatman Grade 3 (WG3) filter paper that was (**a**) “instantaneously” coated and (**b**) coated for thirty minutes in both Aculon™ AL-A and A solutions. (**c**) shows a zoomed-in view of the droplets resting and drying-down on the paper that was coated for an extended time, i.e., thirty minutes. Each substrate contains columns of laser micromachined SETs, where the first column, i.e., left-most, was spotted with 1 microliter (µL) droplets of 0.5% volume/volume green food dye solution (*aqueous*), the second column, i.e., center/middle, was not spotted with a droplet, and the third column, i.e., right-most, was spotted with 1 µL droplets of deionized water. Despite the extended coating time, the SETs in (**b**) and (**c**) did not perfectly confine the droplets, though the performance was better than the SETs prepared on the “instantaneously” coated substrate.

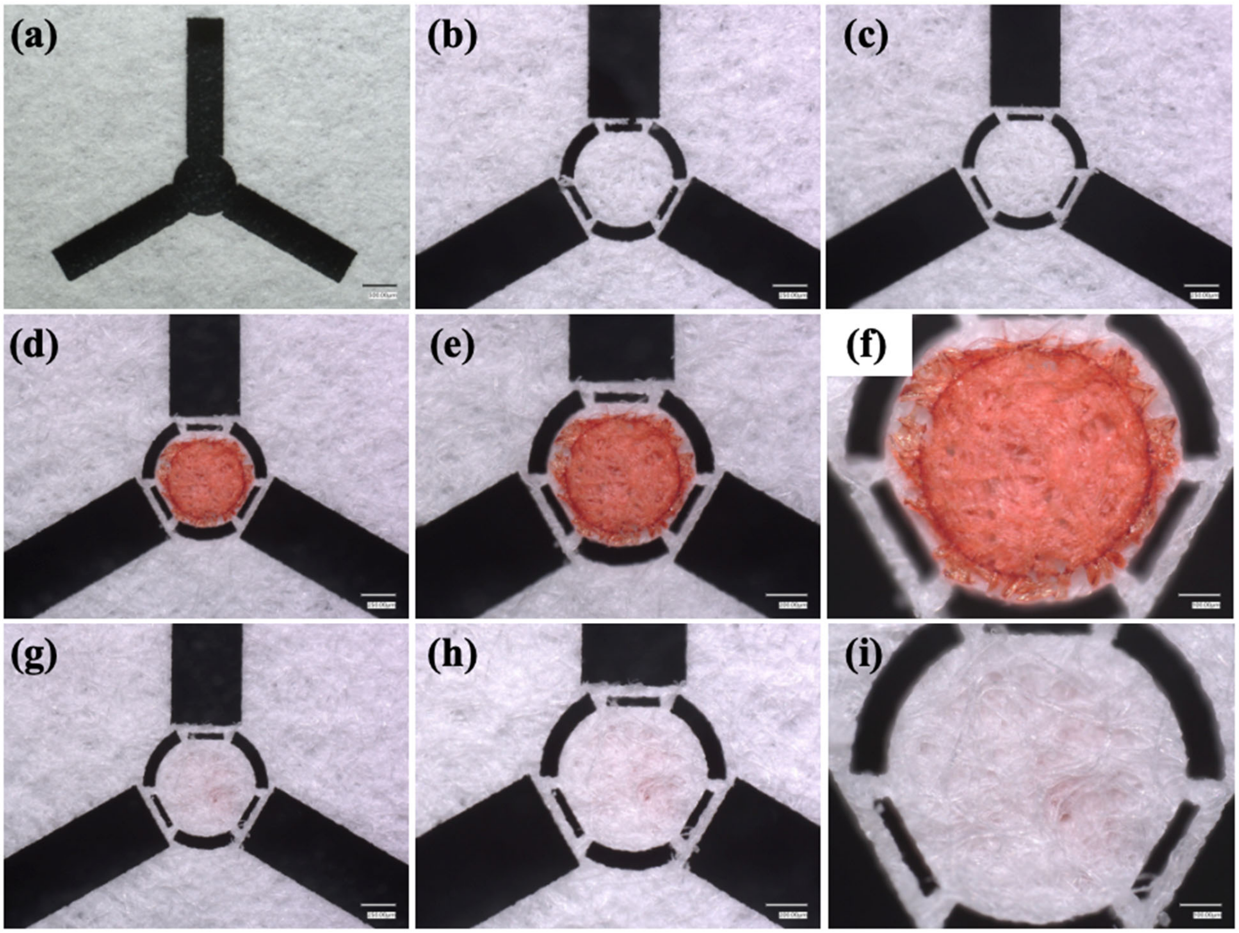

Figure S2. SETs prepared and visualized on Whatman Grade 1 filter paper. (**a**) shows an empty template area where the SET detached. (**b**) shows a partially intact template area where one of the top connecting features has been damaged. (**c**) shows a fully intact, coated template that has been coated but not laser micromachined. (**d–f**) show an increasingly magnified 0.55 mm SET bearing a dried matrix spot resulting from a 1 µL droplet of dilute Allura Red aqueous solution. (**g–i**) show the backside of the same increasingly magnified 0.55 mm SET bearing a dried matrix spot resulting from a 1 µL droplet of dilute Allura Red aqueous solution.

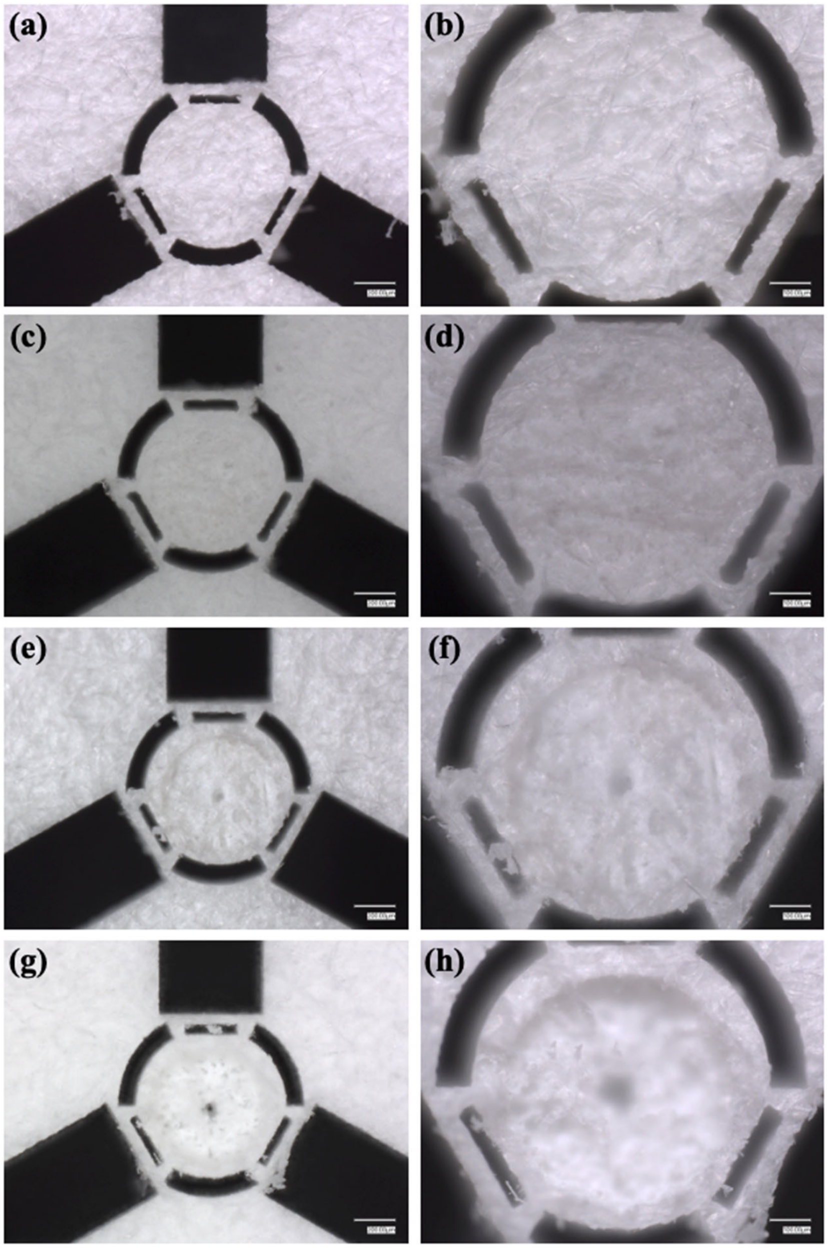

Figure S3. Optical microscopy images of WG3 filter paper bearing SETs prepared at different laser powers. (**a**) and (**b**) show an increasingly magnified coated, non-laser micromachined area where an SET would be prepared, i.e., blank region. (**c**) and (**d**) show an increasingly magnified 0.55 mm diameter SET prepared at 8% laser power. (**e**) and (**f**) show an increasingly magnified 0.55 mm diameter SET prepared at 25% laser power. (**g**) and (**h**) show an increasingly magnified 0.55 mm diameter SET prepared at 50% laser power.

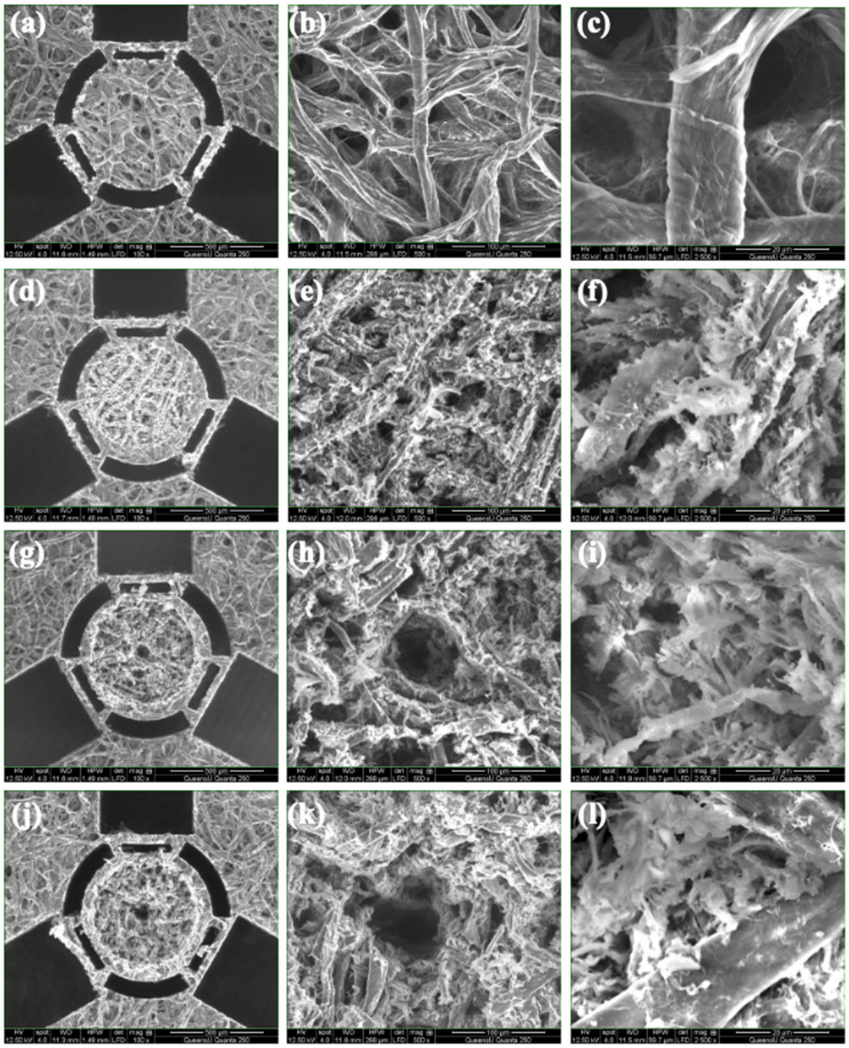

Figure S4. Scanning electron microscopy images of WG3 filter paper bearing SETs prepared at different laser powers. (**a**–**c**) show an increasingly magnified coated, non-laser micromachined area where an SET would be prepared, i.e., blank region. (**d**–**f**) show an increasingly magnified 0.55 mm diameter SET prepared at 8% laser power. (**g**–**i**) show an increasingly magnified 0.55 mm diameter SET prepared at 25% laser power. (**j**–**l**) show an increasingly magnified 0.55 mm diameter SET prepared at 50% laser power.

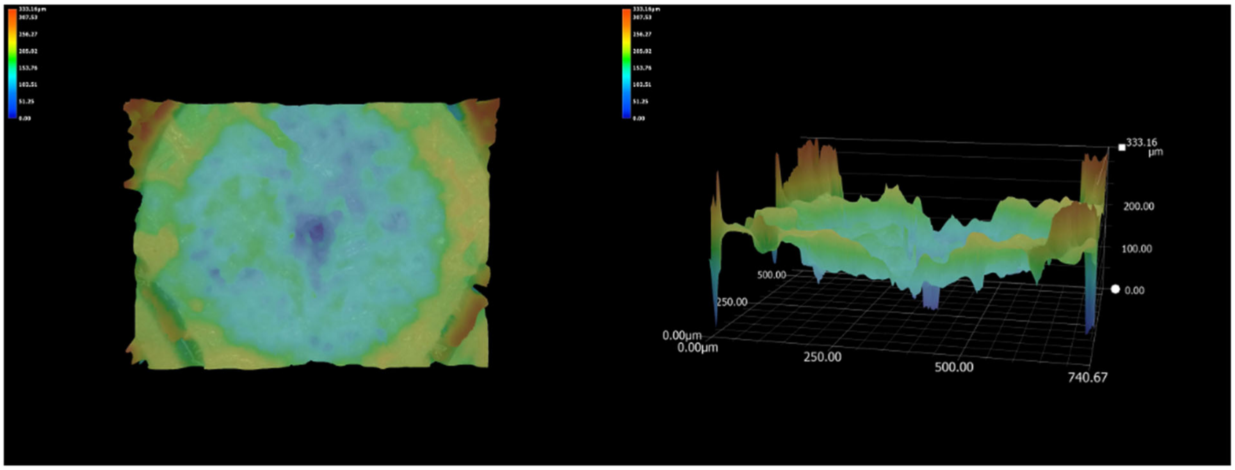

Figure S5. Three-dimensional (3D) depth composition of an SET at 50% laser power.

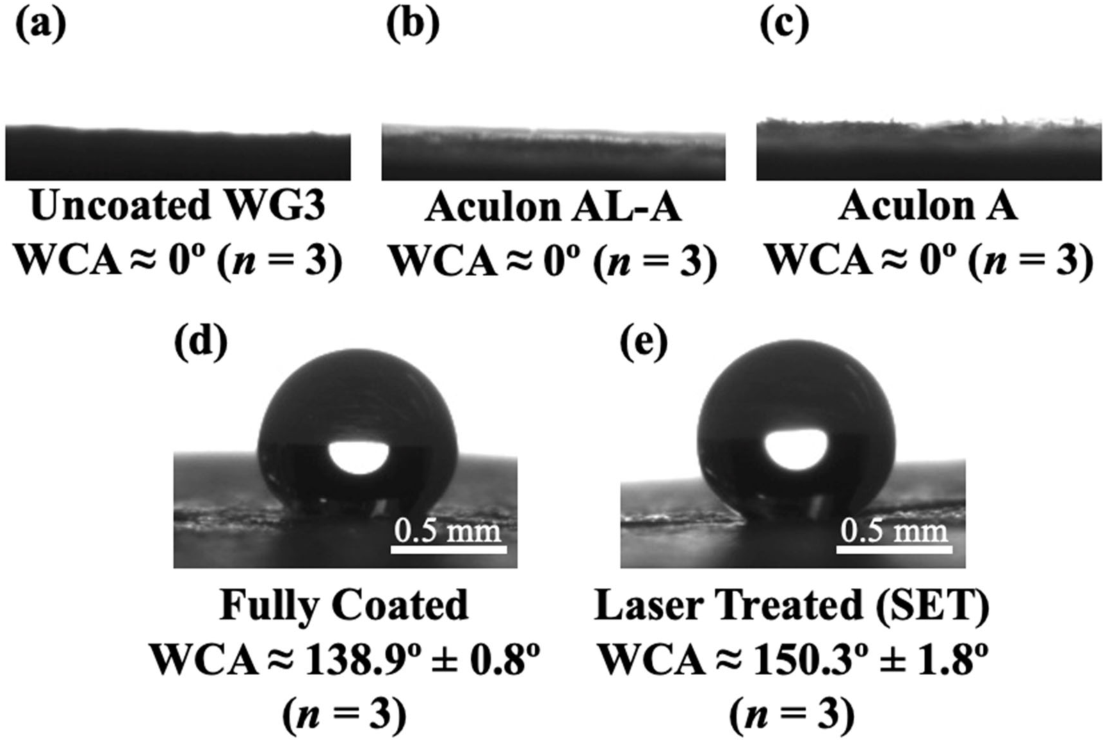

Figure S6. WG3 filter paper at various stages throughout the coating process. (**a**) shows the uncoated filter paper after depositing a 1 μL droplet of deionized water (DI H_2O). (**b**) shows the filter paper coated solely with Aculon AL-A after depositing a 1 μL droplet of DI H_2O. (**c**) shows the filter paper coated solely with Aculon A after depositing a 1 μL droplet of DI H_2O. (**d**) shows the filter paper after the full Aculon coating treatment, i.e., coating with AL-A followed by A, after depositing a 1 μL droplet of DI H_2O. (**e**) shows the fully coated filter paper bearing a laser-micromachined SET after depositing a 1 μL droplet of DI H_2O onto the SET.

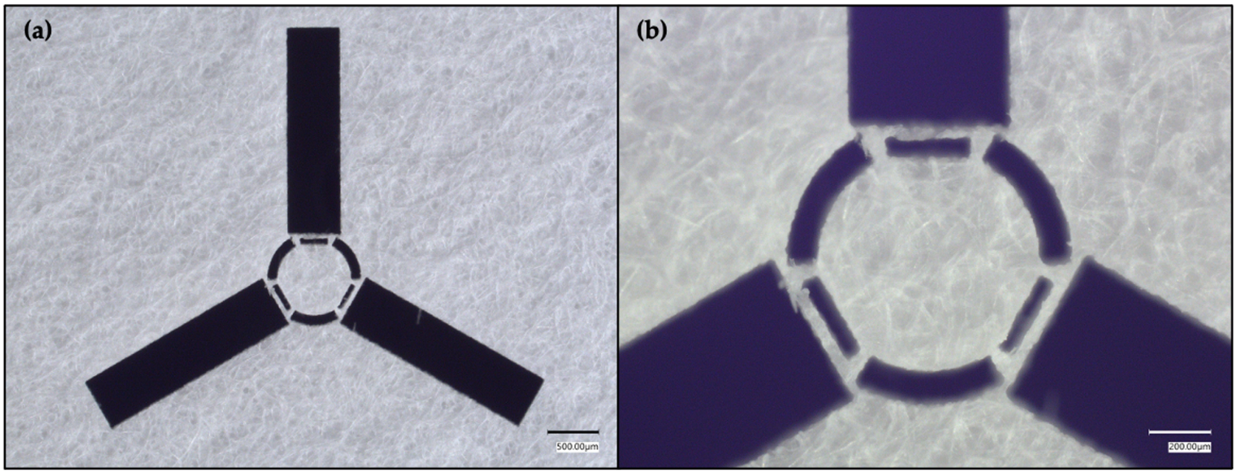

Figure S7. Blank/unspotted targeting SET design with three curved arches and three rectangular excisions. Note that the scale bar in (**a**) represents 500.00 µm and (**b**) represents 200.00 µm.

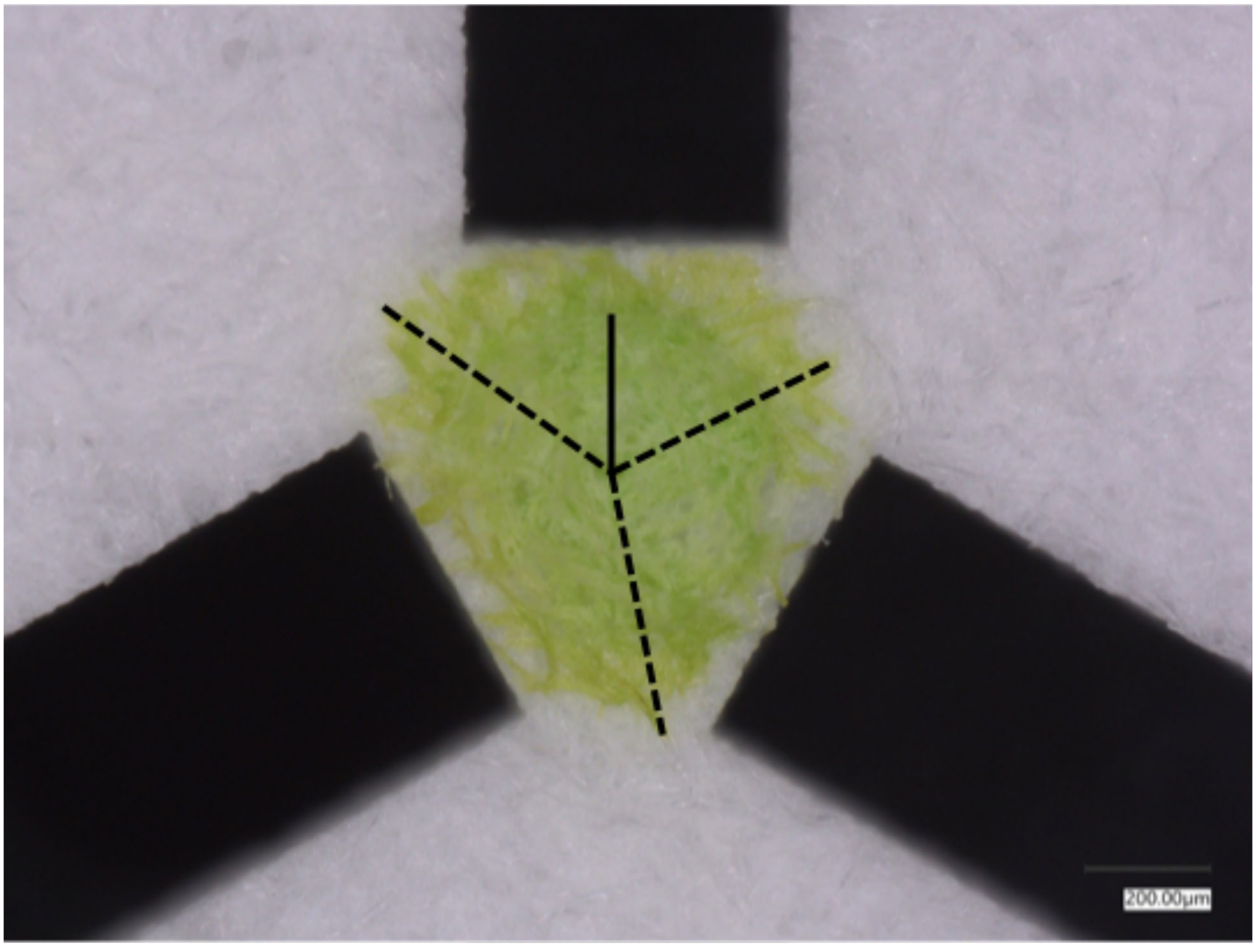

Figure S8. Example showing how the reference line (Solid) was drawn from the center to the edge of the SET and the three "bleed" distance measurement lines (Dashed) were drawn from the center of the SET to three exterior bleeding edges. The reference distance was subtracted from the bleed distance for each of the three lines, and the average distance was taken as the bleed.

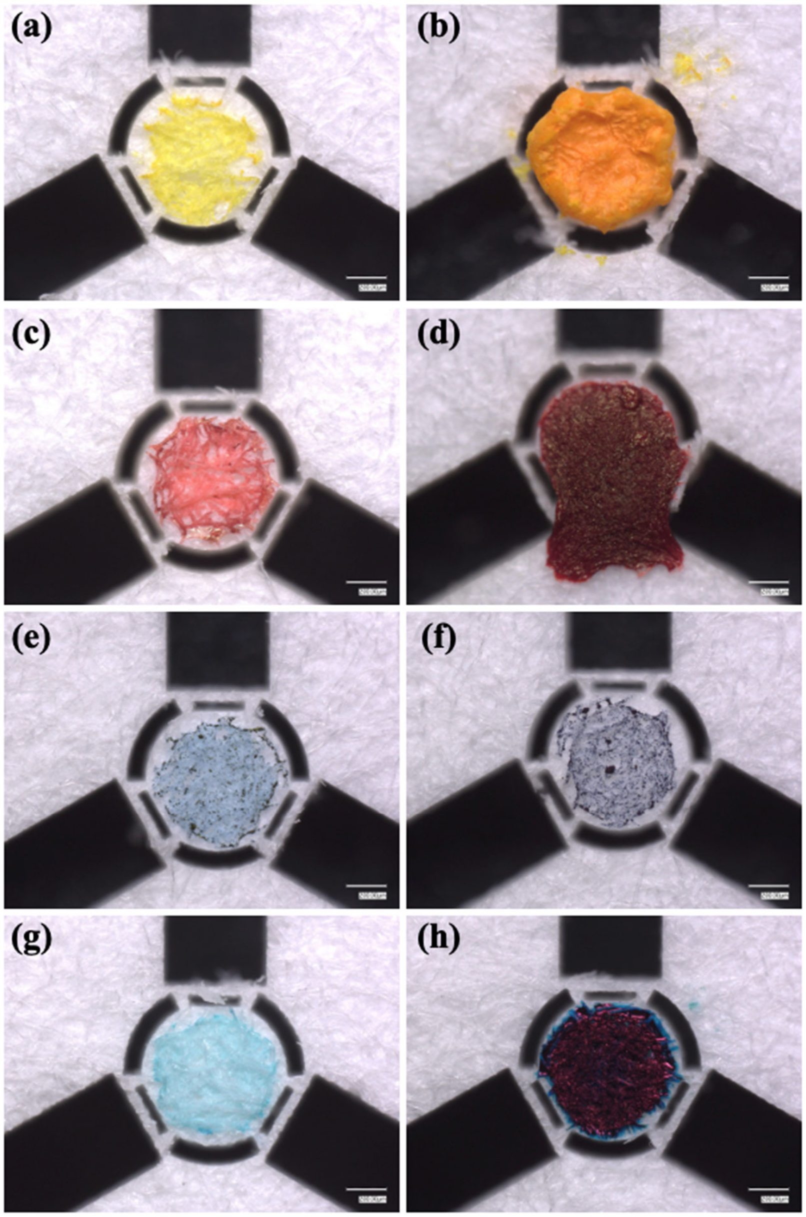

Figure S9. Different visualization agents on 0.55 mm SETs. (**a**) shows the DMS resulting from 1 μL of dilute yellow food dye aqueous solution. (**b**) shows the DMS resulting from 1 μL of undiluted yellow food dye solution. (**c**) shows the dried matrix spot (DMS) resulting from 1 μL of dilute red food dye aqueous solution. (**d**) shows the DMS resulting from 1 μL of undiluted red food dye solution. (**e**) shows the DMS resulting from 1 μL of dilute Nile Blue aqueous solution. (**f**) shows the DMS resulting from 1 μL of a dilute Indigo aqueous suspension (note that Indigo is water insoluble). (**g**) shows the DMS resulting from 1 μL of dilute blue food dye aqueous solution. (**h**) shows the DMS resulting from 1 μL of undiluted blue food dye solution.

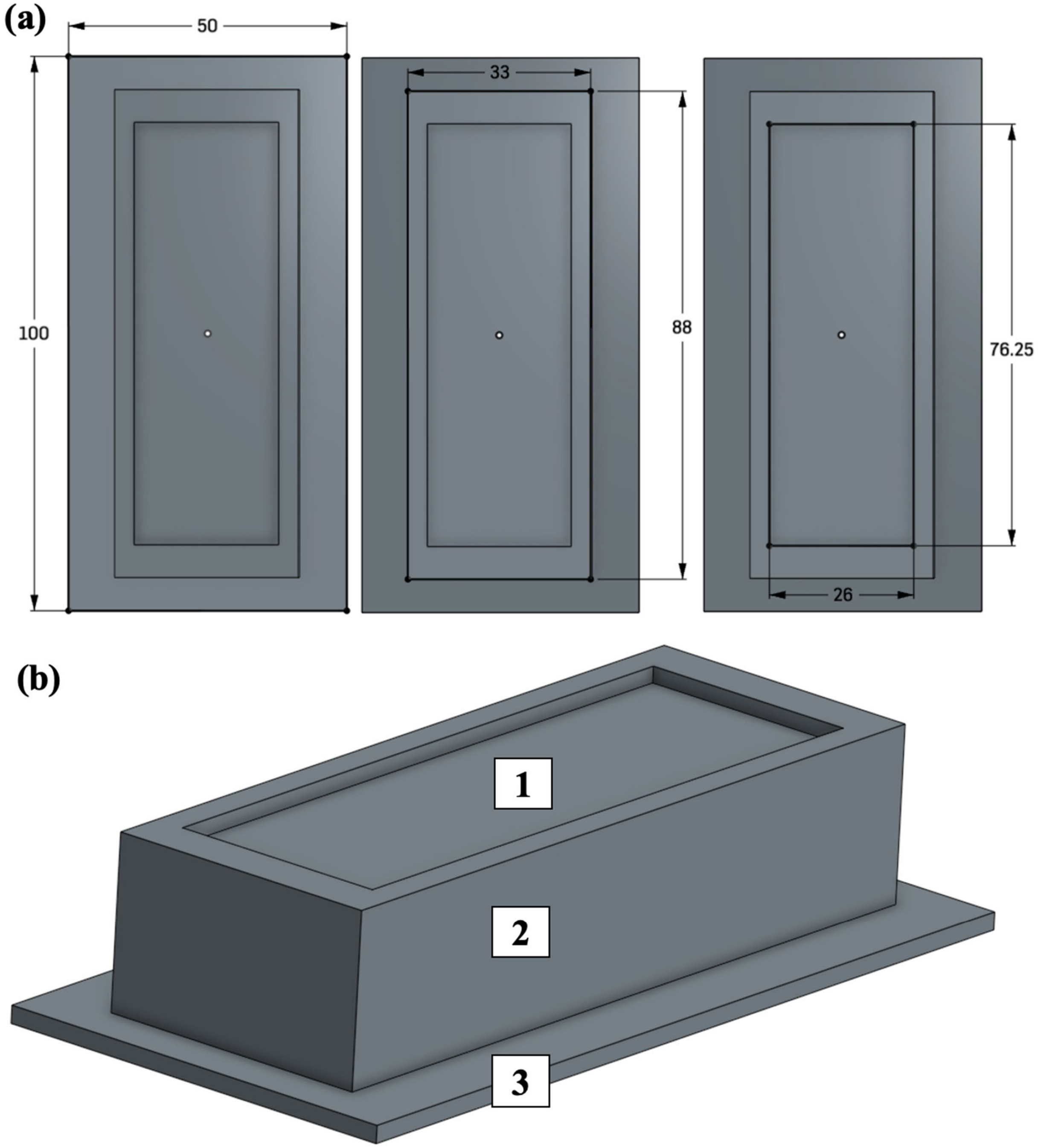

Figure S10. (a) Length/width dimensions of the holder base in millimeters (mm). (b) The heights/thicknesses for the various aspects of the holder base are (1) 1.85 mm (depression/recess where the paper substrate/hydrophobically-coated glass slide are placed), (2) 20 mm, and (3) 2 mm.

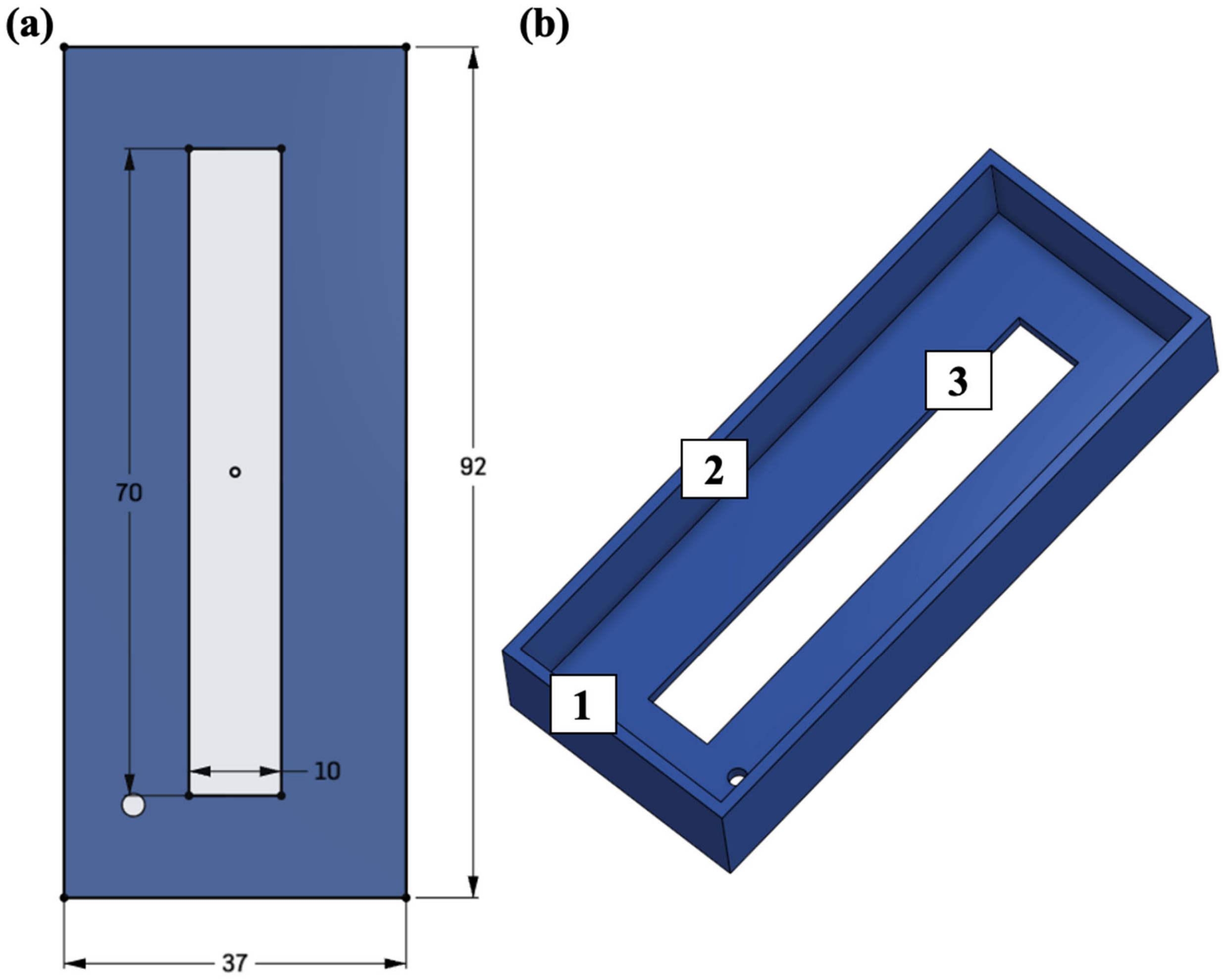

Figure S11. (a) Length/width dimensions of the holder clamp/top in millimeters (mm). (b) The heights/thicknesses for the various aspects of the holder clamp/top are as follows: (1) the wall thickness is 3.25 mm, (2) the walls extend 15 mm from top to bottom, and (3) the sampling window thickness is 2 mm.

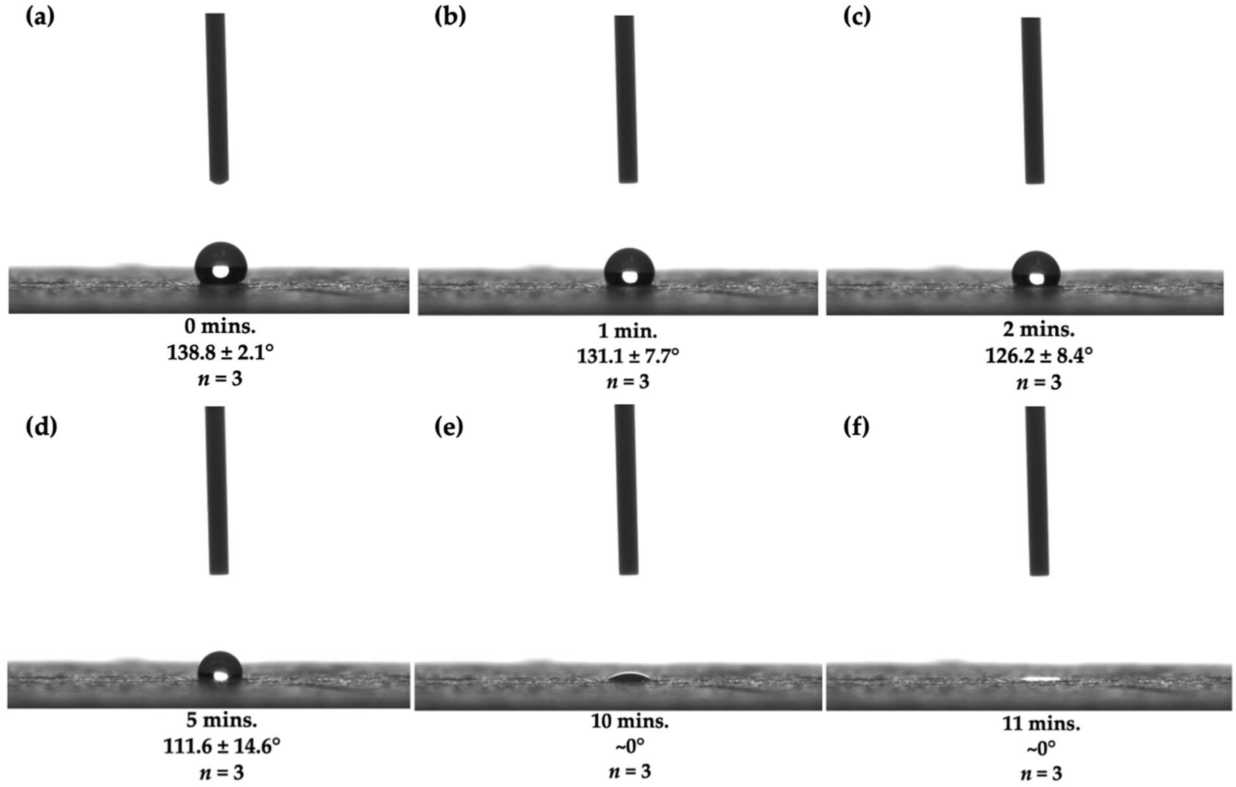

Figure S12. Time lapse of 1 μL deionized water droplet drying-down on paper substrate under ambient conditions. Note that "mins." represents "minutes."

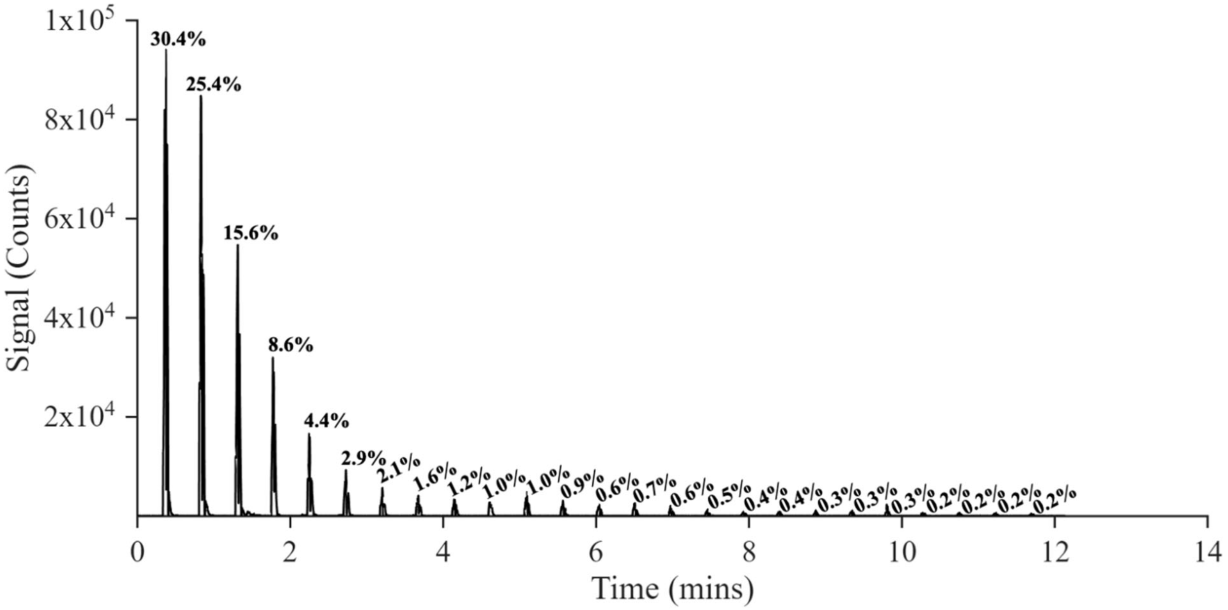

Figure S13. Chromatogram showing caffeine signal depletion of a DMS after repeated sampling by the liquid microjunction–surface sampling probe (LMJ-SSP). The percentages represent the amount of caffeine signal that is collected with each successive sampling by the LMJ-SSP.

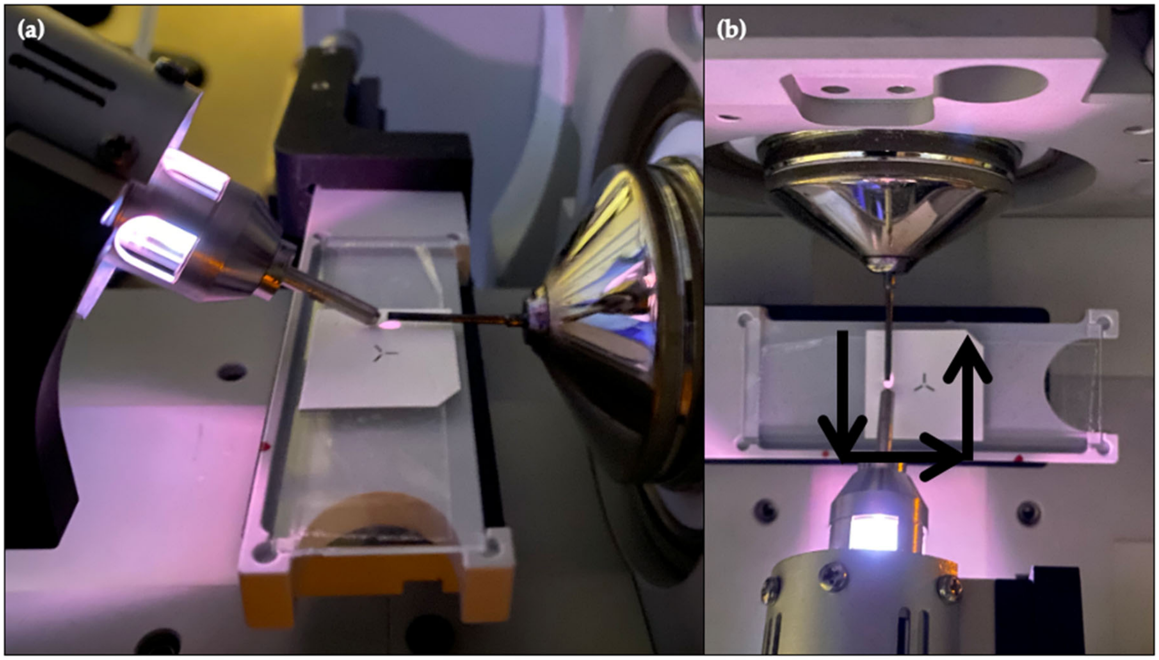

Figure S14. Demonstration of the flowing atmospheric pressure afterglow (FAPA) sampling approach. (**a**) shows the FAPA source idling on a blank area of the substrate prior to the substrate/DMS being “driven”/steered toward the FAPA source/reagent ion stream (note that the FAPA source remains fixed/unmoved). (**b**) shows the movement sequence through which the substrate/DMS is driven toward the ion stream: Following the black arrows from left to right (noting that the arrows refer to the sequential movement/position of the idling plasma visualization spot relative to the substrate), the substrate is moved toward the MS inlet such that the plasma visualization spot appears lower on the substrate; then, the substrate is moved toward the left such that the plasma visualization spot appears more rightwardly, and then the substrate is steadily moved toward/into the plasma visualization spot and thereby into the reagent ion stream. The top crosshair is used to align with the MS inlet during this approach, and movement of the sample holder/substrate/DMS ceases once the SET fully arrives in the sampling location.

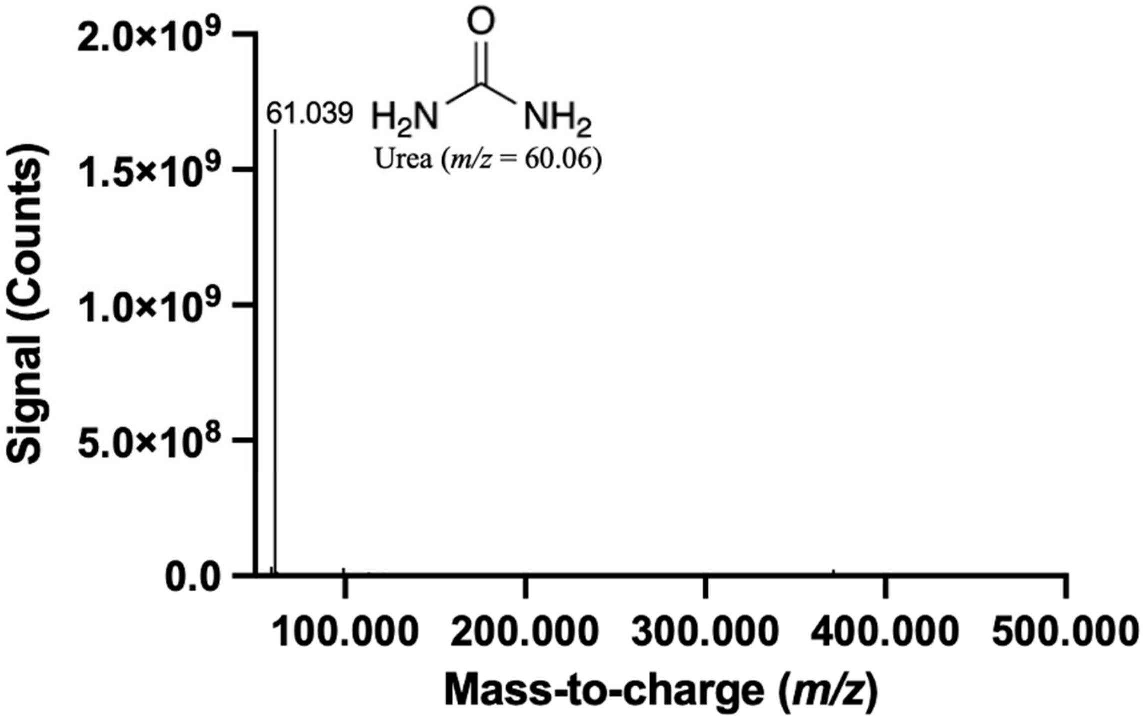

Figure S15. Full-scan spectrum resulting from FAPA-MS analysis of 1 µL of undiluted Pickering Laboratories artificial urine spotted onto targeting SET. The prominent peak at *m/z* 61.039 corresponds to protonated urea.

Video S1. A 1 µL deionized water droplet being pipetted onto uncoated Whatman Grade 1 filter paper.

Video S2. A 1 µL deionized water droplet being pipetted onto uncoated WG3 filter paper.

Video S3. A 1 µL deionized water droplet being pipetted onto hydrophobically coated WG3 filter paper that has been laser micromachined with visual recognition elements flanking an SET.

Video S4. A 1 µL deionized water droplet being deposited onto hydrophobically coated WG3 and held by a syringe needle as the needle is moved horizontally, thereby moving the droplet along the surface horizontally until the SET is encountered, at which point the attractive force of surface energy of the laser-micromachined paper overcomes the droplet's surface tension; the droplet can be observed separating from the mother liquid/syringe tip and remaining on the SET as the needles continues to move horizontally.

Video S5. A 1 µL droplet of deionized water colored with green food dye being pipetted onto hydrophobically coated WG3 filter paper that has been laser micromachined with visual recognition elements flanking SETs; the SETs were prepared using various increasing amounts of laser power with constant laser speed, ranging from 10% to 40% laser power (see also graphic guide below for reference, where 80X represents the video playback speed).

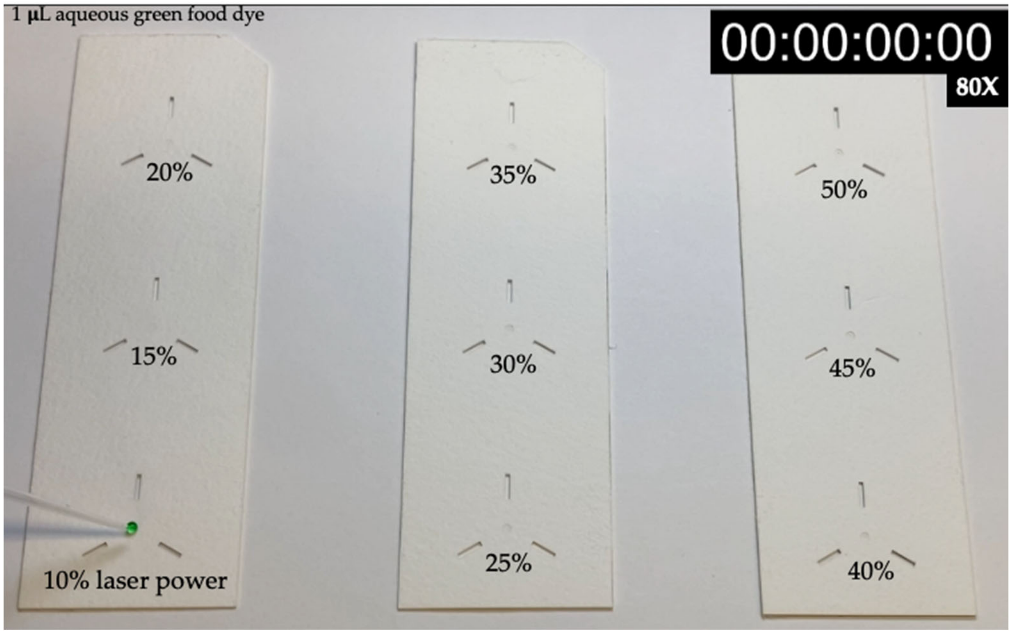

Video S6. Several 1 µL droplets of deionized water colored with green food dye being pipetted onto hydrophobically coated WG3 filter paper that has been laser micromachined with visual recognition elements flanking SETs; the SETs were prepared using various increasing amounts of laser speed with constant laser power, ranging from 1.0 millimeters per second (mm/s) to 4.0 mm/s laser speed (see also graphic guide below for reference, where 100X represents the video playback speed).

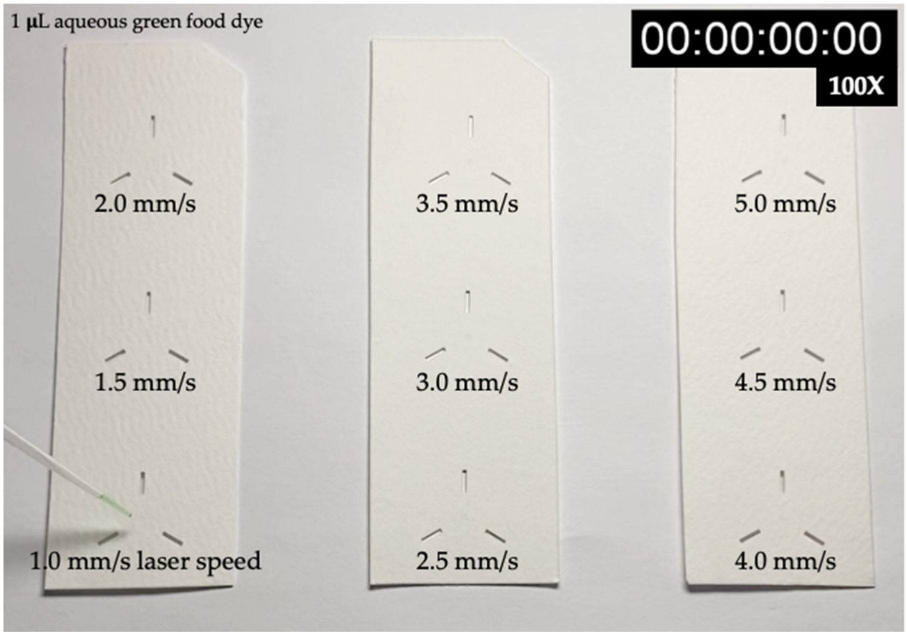

Video S7. Demonstration of the modified 3D printer chassis controlling the LMJ-SSP for automated sampling.
